# Marine *Streptomyces* sp. derived antimycin analogues suppress HeLa cells via depletion HPV E6/E7 mediated by ROS-dependent ubiquitin–proteasome system

**DOI:** 10.1038/srep42180

**Published:** 2017-02-08

**Authors:** Weiyi Zhang, Qian Che, Hongsheng Tan, Xin Qi, Jing Li, Dehai Li, Qianqun Gu, Tianjiao Zhu, Ming Liu

**Affiliations:** 1 Key Laboratory of Marine Drugs, Chinese Ministry of Education, School of Medicine and Pharmacy, Ocean University of China, Qingdao 266003, People’s Republic of China; 2 Laboratory for Marine Drugs and Bioproducts of Qingdao National Laboratory for Marine Science and Technology, 266237, People’s Republic of China

## Abstract

Four new antimycin alkaloids (**1**–**4**) and six related known analogs (**5**–**10**) were isolated from the culture of a marine derived *Streptomyces* sp. THS-55, and their structures were elucidated by extensive spectroscopic analysis. All of the compounds exhibited potent cytotoxicity *in vitro* against HPV-transformed HeLa cell line. Among them, compounds **6**–**7** were derived as natural products for the first time, and compound **5** (NADA) showed the highest potency. NADA inhibited the proliferation, arrested cell cycle distribution, and triggered apoptosis in HeLa cancer cells. Our molecular mechanic studies revealed NADA degraded the levels of E6/E7 oncoproteins through ROS-mediated ubiquitin-dependent proteasome system activation. This is the first report that demonstrates antimycin alkaloids analogue induces the degradation of high-risk HPV E6/E7 oncoproteins and finally induces apoptosis in cervical cancer cells. The present work suggested that these analogues could serve as lead compounds for the development of HPV-infected cervical cancer therapeutic agents, as well as research tools for the study of E6/E7 functions.

Cervical cancer is the second most common malignancy among women, which is primarily resulted from humanpaplilloma virus (HPVs) infections, such as HPV-16 and HPV-18^1^. Intergradation of the HPV viral genome into the host cells and the expression of viral proteins E6/E7 contribute to carcinogenesis and malignant growth of cervical cancer^2^. In cervical cancer, oncoproteins E6/E7 modulate key cell signaling components. E6 binds to cancer suppressor p53 and facilitates the degradation and dysfunction of p53^3^. E7 interacts with retinoblastoma protein (Rb), causes the proteolytic degradation of Rb, and stimulates the cell cycle progression^4^. Moreover, E6 can activate phosphoinositide 3-kinase/protein kinase B (PI3K/Akt) pathway^5^ and mammalian target of rapamycin (mTOR)^6^; over-expression of E6/E7 can increase the level of phosphorylated extracellular signal-regulated kinase (Erk1/2) in cancer cell lines^7^. Other study also showed that the expression level of E6/E7 can be decreased by suppressing expression of the signal transducer and activator of transcription 3 (STAT3)^8^. Physiologically, HPV E6/E7 oncoproteins are degraded by the ubiquitin-dependent proteasome system (UPS). In this degradation pathway, E6/E7 are first ubiquitinated, and the ubiquitinated E6/E7 are then degraded by the proteasome^9^,^10^,^11^,^12^. Ubiquitination and proteasomal degradation of the UPS substrates, *e.g.* E6/E7, have a close relationship with reactive oxygen species (ROS). ROS inducers can increase the enzymatic activities of the ubiquitin-conjugation system, and enhance the level of Ub-conjugates^13^,^14^, while ROS scavenges can impair the proteasome activity in cancer cells^15^.

The sustained activation of cancer cell survive signaling through p53 and Rb, makes high-risk HPV E6/E7 oncoproteins potential drug targets for the treatment of cervical cancer^16^. In recent years, natural compounds have been extensively explored for their potential usage as treatments for cervical cancer via suppressing E6/E7. For example, Tanshinone IIA inhibited E6/E7 at the transcription level and led to the reactivation of the p53-dependent growth inhibition of cervical cancer cells^17^; Anisomelic acid down-regulated E6/E7 at protein level by proteasome degradation and induced p53-independent mitochondrial apoptosis in Siha cells^18^; N-benzylcinnamide, purified from Piper submultinerve, promoted human cervical carcinoma CaSki and HeLa cell lines apoptosis by inhibiting E6/E7 expression at mRNA level^19^; Docosahexaenoic acid caused ROS-dependent-UPS-mediated E6/E7 degradation and the death of HPV-associated cancer cells^20^. Thus, natural compounds which induce E6/E7 degradation could be a remarkable source of anti-cervical cancer agents.

We have studied the bioactive secondary metabolites from marine-derived actinomycetes^21^. *Streptomyces* sp. strain THS-55, was isolated from the marine sediment collected at the mangrove conservation in Hainan province, China. The crude extract of THS-55 showed a significant cytotoxicity against HeLa cells (with an inhibitory rate 79.4% at the concentration of 100 μg/mL) and an interesting HPLC profile. Further analysis of the crude extract led to the isolation and structural elucidation of four new antimycin alkaloids (**1–4**) and six known analogues including N-acetyl-deformylantimycin A (**5,** termed as NADA), deformylated antimycin A_2a_ (**6**), deformylated antimycin A_1a_ (**7**)^22^, antimycin A_18_ (**8**)^23^, antimycin A_6a_ (**9**)^24^ and antimycin A_4a_ (**10**)^25^. Compounds **6** and **7** were identified as natural products for the first time. The chemical structures of these isolated compounds are shown in Fig. 1. Herein, we reported the isolation, structural determination, and cytotoxicity of these compounds. These studies focused on NADA with special emphasis to its cytotoxicity against HeLa cells and its effect on viral oncogenges E6/E7. Our results showed that NADA inhibited cell proliferation, arrested cell cycle, triggered apoptosis, and down-regulated E6/E7 through ROS-mediated UPS activation in HeLa cells. This is the first report that demonstrates antimycin-type analogue induces E6/E7 oncoproteins degradation via stimulation on UPS and finally induces apoptosis in HPV positive cervical cancer cells.

## Results

### Structure elucidation of antimycin analogues

**Figure 1:**
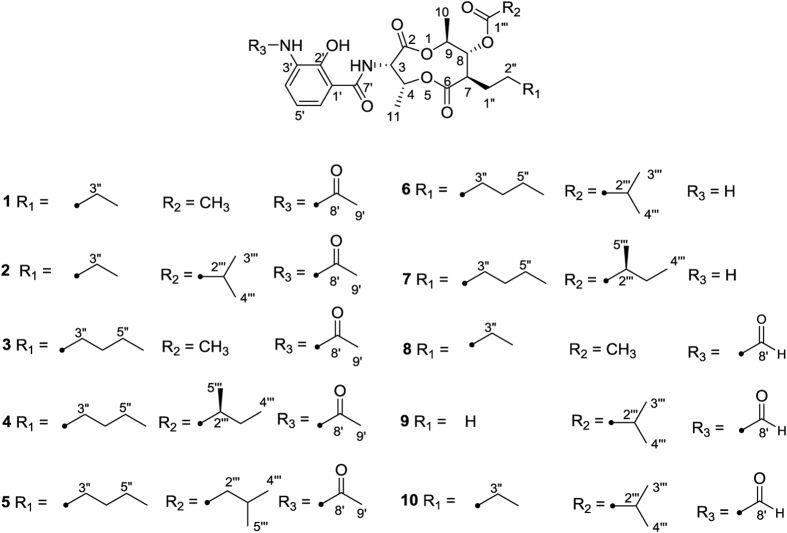
Structures of compounds **1–10**.

**Figure 2:**
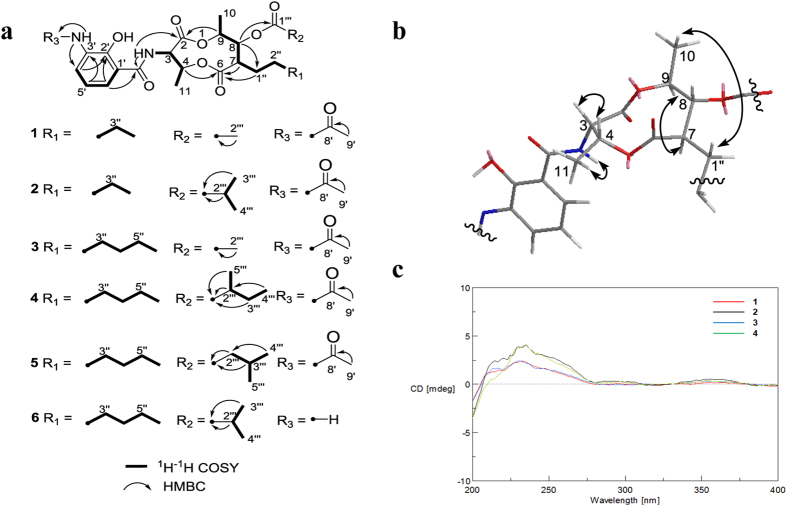
Spectroscopic data of the isolated compounds. (**a**) The Key ^1^H-^1^H COSY and HMBC correlations of **1**–**6**. (**b**) Key NOESY correlations of **1**–**4**. (**c**) Experimental ECD spectra of compounds **1**–**4**.

The molecular formula of compound **1** was established as C_24_H_32_N_2_O_9_ based upon the observation of HRESIMS ion peak at *m/z* 493.2189 [M + H]^+^ (calcd for C_24_H_33_N_2_O_9_, 493.2181). The 1D NMR data suggested the presence of five methyls, three methylenes, eight methines and eight quaternary carbons. The ^1^H and ^13^C NMR data (Supplementary Tables S1 and S2) were very similar to those of antimycin A_18_^23^ except for the presence of an acetyl group in **1**, instead of the aldehyde group in antimycin A_18_, which was further confirmed by the HMBC correlation from H_3_–9′ (*δ*_H_ 2.23) to C-8′ (*δ*_C_ 168.7) (Fig. 2). Therefore, the structure of **1** was elucidated, as shown in Fig. 1, as a new member of antimycin family, named antimycin E.

Antimycins F (**2**) and G (**3**) have the same molecular formula C_26_H_36_N_2_O_9_, which were established on the basis of the HRESIMS analysis of ions at *m/z* 521.2487 [M + H]^+^ and 521.2482 [M + H]^+^, respectively. Compounds **2** and **3** were isolated as a mixture with the ratio (3:1). These two compounds were further separated by a chiral-phase column (Chiralpak IB). Comparison of the NMR spectra of **2** with those of **1** revealed that the acetyl group attached to 8-O in the structure of **1** was replaced by an isobutyryl group in **2**, which was further confirmed by analysis of the ^1^H-^1^H COSY and HMBC correlations of **2** (Fig. 2a). Analysis of the NMR data of **3**, in comparison to **1** revealed that the alkyl chain at C-7 was longer by two methylenes, which was further confirmed by analysis of the ^1^H-^1^H COSY correlations of **3** (Fig. 2a).

Antimycins H (**4**) and NADA (**5**) were also obtained as a mixture by a C18 semi-preparative HPLC column. Both of them were assigned the same molecular formula C_29_H_42_N_2_O_9_ on the basis of the HRESIMS analysis (*m/z* 563.2947 [M + H]^+^ and 563.2950 [M + H]^+^, respectively). The ^1^H and ^13^C NMR spectra of new compound **4** was very similar to those of antimycins A_1a_^25^, except for the presence of an acetyl group (*δ*_H_ 2.24, *δ*_C_ 24.9), which was confirmed by the HMBC correlation (Fig. 2a) from H_3_-9′ (*δ*_H_ 2.24) to C-8′ (*δ*_C_ 168.7). Thus, the structure of **4** was determined as the acetylated version of antimycins A_1a_. Compounds **4** and **5** were separated from their mixture using a chiral-phase HPLC column (Chiralpak IA).

The relative configurations of **1–4** were established by analysis of proton coupling constants and NOESY data. The *syn* configuration of H-3 and H-4 was deduced by the coupling constants (^3^*J*_H-3, H-4_ = 5.90–7.40 Hz) and the NOESY correlations between H-3 and H-4, and between NH and H3–11 (Fig. 2b). The large coupling constants (9.80–10.55 Hz) between H-7/H-8, H-8/H-9 in each of compounds **1–4** indicated an *anti* relative configuration of those protons. Thus, H-7 and H-9 had a *syn* relationship, which was supported by the NOESY correlations of H-9/H-7 and H-10/H-1” (Fig. 2b).

To determine the absolute configurations of **1**–**4**, their CD spectra were compared with that of known compounds^26^. They all showed a weak positive Cotton effects (CE) at 230 nm dominated by the spatial position of the 3-acetamido-2-hydroxybenzamide moiety (Fig. 2c), which was in accordance with a reported 8-hydroxy antimycin^26^. In addition, the optical rotations of **1**–**4** are very similar to known antimycins^23^. These data, combined with the NMR data of the nine-membered dilactones of **1–4**, provide strong evidence that **1–4** have the same absolute configurations as known antimycins. Thus, the absolute configurations of the nine-membered dilactone ring of **1**–**4** were assigned as 3*S*, 4*R*, 7*R*, 8*R*, and 9*S*. The *S*-configuration of C-2″′ in compound **4** was tentatively assigned by the comparison of the chemical shifts of 5″′-CH_3_ (*δ*_H_ 1.190) with those of the synthetic 2″′*S*-antimycin A_3a_ (*δ*_H_ 1.190) and 2″′*R*-antimycin A_3a_ (*δ*_H_ 1.197) which contained the same isovaleric acid side chain^27^.

The deformylated antimycins, compounds **6**–**7**, had been previously obtained artificially by treating the antimycins with either hot hydrochloric acid or diisobutylaluminum hydride^28^. In this study, they were for the first time isolated from a natural source and provided the spectroscopic data.

### NADA is cytotoxic and arrests HeLa cells at S phase

**Figure 3:**
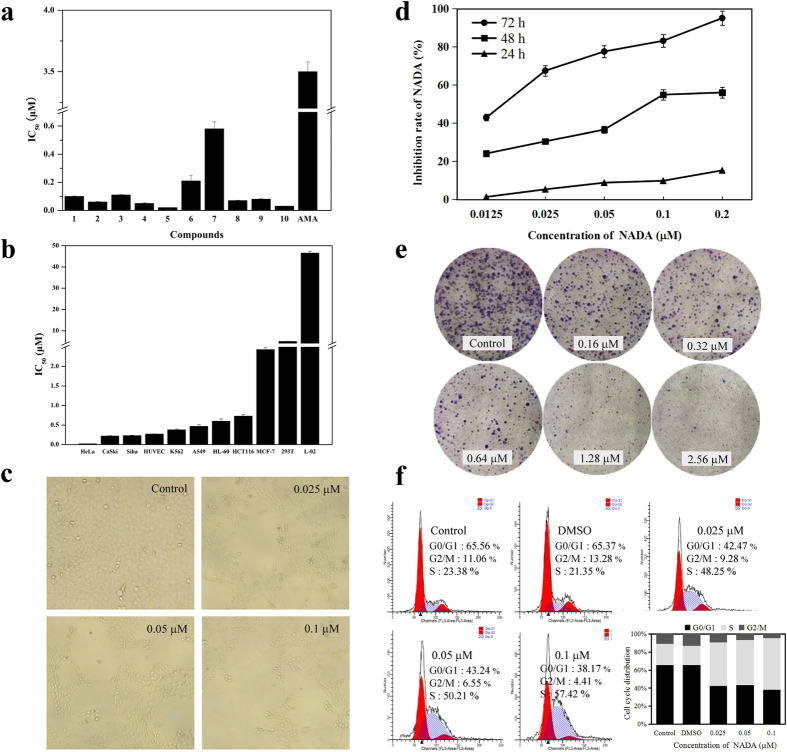
Cytotoxicity of compound **1**–**10** and the effect of NADA on HeLa cells. (**a**) The cytotoxicity of **1**–**10** as well as AMA was evaluated against HeLa cell line. Cells were treated with **1**–**10** for 72 h, then the cell viability were assayed by 3-(4, 5-dimethylthiazol-2-yl)-2, 5-diphenyltetrazolium bromide (MTT) methods. (**b**) NADA displayed selective cytotoxicity against HPV-positive cell lines. The selected cell lines were treated with different concentrations of NADA for 72 h, and the cell viability were assayed by MTT methods. (**c**) Representative pictures showing the effect on cell density of 0, 0.025, 0.05, and 0.1 μM NADA, after incubation for 72 h. (**d**) NADA inhibited proliferation of HeLa cells time- and concentration- dependently, assayed by MTT method. (**e**) Results of colony-formation assays of HeLa cells after NADA (0–2.56 μM) treatment for 12 days, with Giemsa staining. (**f**) NADA induced S phase arrest in HeLa cells. Histogram shows the percentage of cells in G0/G1, G2/M, and S phase, after treated with NADA. All experiments were performed in three replicates (n = 3).

Compound **1–10** exhibit different cytotoxicity against HeLa cell line. Most of their IC_50_ values were in the nanomolar range with better activity than the commercial antimycin A (AMA) (Fig. 3a). NADA and compound **10** were much more potent than the other compounds, with IC_50_ values of 0.02 and 0.03 μM, respectively. Compound **6** and **7** had the weakest potency against HeLa cells. Structure-activity relationship (SAR) analysis indicated that the acyl group (formyl or acetyl) attached to 3′-NH might be important for HeLa cytotoxicity, and the poor activity of compound **6** and **7** was because these are amine at physiological pH ammonium ions. Furthermore, a long group at R2 was clearly good for bioactivity and R1 seemed less important. Considering its potent activity against HeLa cell line, we selected NADA, the most potent compound against HeLa cells, to perform detailed cytotoxicity and mechanism studies. Besides NADA, other isolated compounds also showed different cytotoxicity towards other selected cell lines (Supplementary Table S3).

To evaluate the cytotoxicity range of NADA, we tested its effect on other cell lines, including HPV-positive cervical cancer CaSki and Siha cell lines, HPV-negative cancer A549, MCF-7, HCT116, K562, and HL-60 cell lines, human umbilical vein endothelial HUVEC cell line, and normal cell lines (human normal liver cells L-02 and embryonic kidney cells 293 T). Interestingly, NADA also showed relative more potent cytotoxic effect against HPV-positive Siha and CaSki cells, while less cytotoxicity towards HPV-negative cell lines (Fig. 3b). The cytotoxic effect of NADA against these HPV-positive cervical cancers was much stronger than the clinically used drugs, including cisplatin, imatinib, sorafenib, and taxol (Supplementary Table S4). Treatment with NADA clearly decreased HeLa cells population (Fig. 3c). The IC_50_ values of NADA against HeLa cells were 1.22, 0.11, and 0.02 μM after 24, 48, and 72 h treatment, respectively, presenting a concentration- and time-dependent manner (Fig. 3d). The cytotoxicity of NADA was also confirmed by the colony-forming assay on HeLa cells (Fig. 3e). In order to explore whether the cell cycle arrest contributed to NADA-induced proliferation inhibition, we further analyzed the cell cycle distribution, and we found that NADA induced S phase arrest in HeLa cells in a concentration dependent manner (Fig. 3f).

### NADA induces caspases-dependent apoptosis in HeLa cells

**Figure 4:**
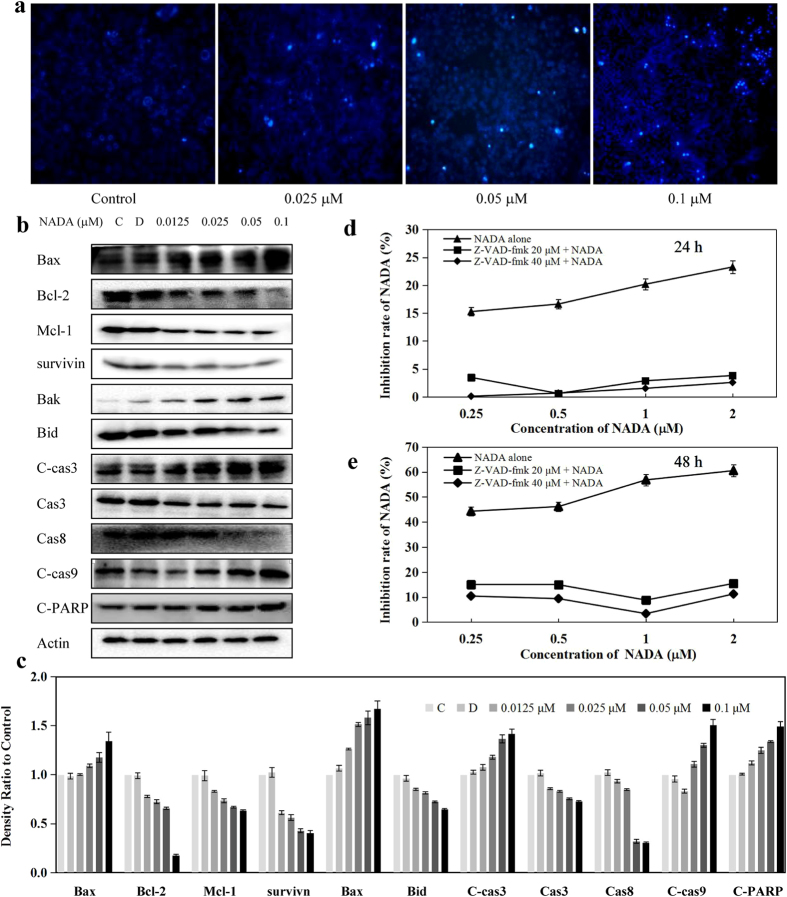
NADA induced HeLa cells apoptosis. (**a**) HeLa cells were treated with indicated concentration of NADA for 24 h, stained by Hoechst 33342, and observed by fluorescence micrograph. (**b**) Effect of NADA on apoptosis-related proteins. HeLa cells were treated with indicated concentrations of NADA for 24 h, and the apoptosis-related proteins were detected by western blotting. (**c**) Histograms show the intensity of apoptosis-related protein bands. HeLa cells were treated with caspase inhibitor Z-VAD-fmk for 1 h, and then NADA for 24 h (**d**) and 48 h (**e**). Inhibition of NADA was measured by MTT assays. Values represent the means ± SD. *P < 0.05, **P < 0.01 versus control. All experiments were performed in three replicates (n = 3).

In order to analyze NADA stimulated apoptosis in HeLa cells, we monitored the morphological changes of nuclear chromatins after Hoechst 33342 staining. As shown in Fig. 4a, the number of cell nuclei that exhibited brighter blue fluorescence increased significantly after exposure to NADA, indicating the induction of apoptosis. Furthermore, we observed, as shown in Fig. 4b,c, the decrease in the anti-apoptotic proteins including Bcl-2, Mcl-1, Bid and survivin, as well as the increase in pro-apoptotic proteins Bax and Bak in the presence of NADA. In addition, after NADA treatment, caspase 8 and caspase 3 were decreased, cleaved-caspase 3, cleaved caspase 9 and cleaved PARP (C-PARP) were increased. These results further confirmed the occurrence of apoptosis. When pretreated with pan caspase inhibitor Z-VAD-FMK, cells became insensitive to NADA (Fig. 4d,e), which suggested caspases were involved in NADA-induced apoptosis. All these results demonstrated that NADA could induce caspases-dependent apoptosis in HeLa cells.

### NADA disrupts mitochondrial membrane potential (MMP) and inhibits mitochondrial respiration

**Figure 5:**
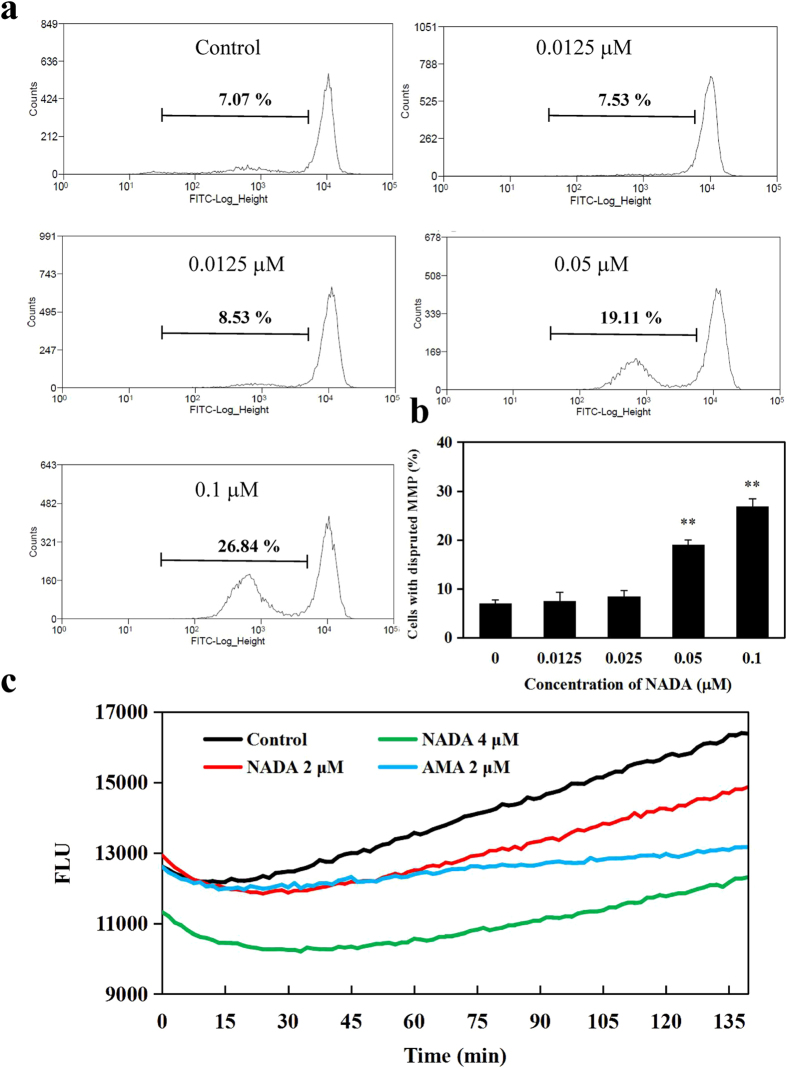
NADA induced the loss of MMP in HeLa cells. (**a**) HeLa cells were treated with indicated concentration of NADA for 24 h, stained with Rho 123, and detected by flow cytometry. (**b**) Histogram shows the percentage of cells with disrupted MMP after treated with NADA. (**c**) NADA inhibited the extracellular O_2_ consumption and disrupts the mitochondrial respiration of HeLa cells. Values represent the means ± SD. **P < 0.01 versus control. All experiments were performed in three replicates (n = 3).

MMP is an important indicator of mitochondrial function and close related to apoptosis. We then tested the effect of NADA on MMP using Rhodamine 123 (Rho 123) probe. The percentage of the depolarized mitochondria was about 7% in the control group. When exposure to 0.05 and 0.1 μM NADA, the percentage of cells with depolarized MMP elevated to 19.11 and 26.84%, respectively, in a concentration-dependent manner (Fig. 5a,b), suggesting that NADA could disrupt the mitochondrial function of HeLa cells. Furthermore, we detected the extracellular O_2_ consumption and mitochondrial respiration of HeLa cells in the presence of NADA. We found that, as the positive control AMA, NADA could inhibit the extracellular O_2_ consumption and subsequently disrupt the mitochondrial respiration of HeLa cells (Fig. 5c).

### NADA degrades HPV E6/E7 viral oncoproteins and inhibits its function

**Figure 6:**
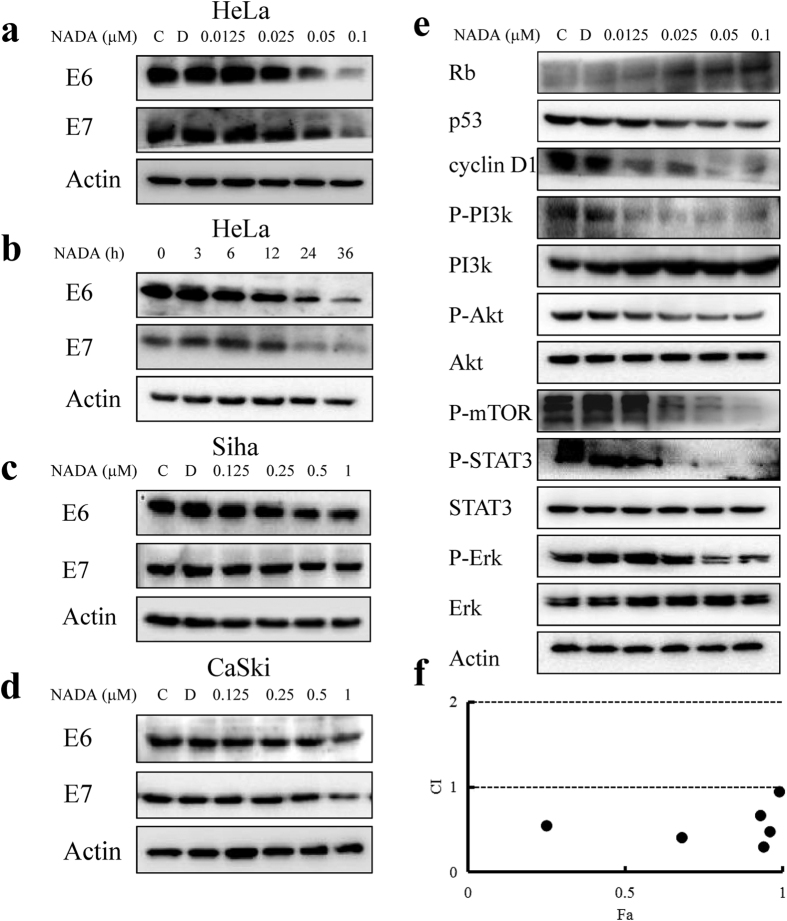
NADA induced E6/E7 viral oncoproteins degradation and depresses the function of E6/E7. (**a**) HeLa cells were treated with the indicated concentrations of NADA for 24 h. The expression levels of E6/E7 were assessed by western blotting. (**b**) HeLa cells were incubated with 0.1 μM NADA for the indicated times. The expression levels of E6/E7 were assessed by western blotting. (**c**) NADA induced E6/E7 degradation in Siha cells. **(d**) NADA induced E6/E7 degradation in CaSki cells. (**e**) HeLa cells were treated with the indicated concentrations of NADA for 24 h, and the E6/E7 relevant signaling molecules were assessed. Histograms show the quantity analyses for intensity of these bands in a-e are shown as Supplementary Fig. S2. (**f**) NADA showed synergistic effect with cisplatin. The combination index (CI) reflecting the synergism of two drugs was calculated using software package Calcusyn (Biosoft, Cambridge, UK). The CI values of < 1, 1, and > 1 indicate synergistic, additive, and antagonistic effects, respectively. All experiments were performed in three replicates (n = 3).

Considering HPV E6/E7 play a key role in the survival and the malignant phenotype of HPV-infected cancer cells, such as HeLa^29^, we further examined the effect of NADA on E6/E7. Our results showed that NADA significantly reduced the expression levels of E6/E7 in concentration-dependent (Fig. 6a) and time-dependent (Fig. 6b) manners. Similarly, NADA could decrease the level of E6/E7 in HPV-positive Siha (Fig. 6c) and CaSki (Fig. 6d) cell lines. However, NADA treatment did not cause significant down-regulation of neither E6 nor E7 mRNA expression level (Supplementary Fig. S1). As the primary cellular targets of E6/E7, p53 and Rb, would restore their own function after E6/E7 suppression^30^. Inhibition of E6 led to increased p53, while inhibition of E7 resulted in increased Rb^31^. We therefore measured the expression level of p53 and Rb. As expected, Rb increased after treatment with increasing concentration of NADA. Surprisingly, p53 decreased remarkably (Fig. 6e). As the downstream molecule of E6, cyclin D1 can be upregulated by E6^32^. In our results, cyclin D1 expression level decreased obviously in the presence of NADA (Fig. 6e). E6/E7 activates PI3K/Akt/mTOR pathway^6^,^33^,^34^, Erk1/2 pathway^35^, and STAT3 pathway^8^,^36^. These activated pathways in turn promote cell proliferation, avoid apoptosis, and decrease chemical drug sensitivity. NADA treatment remarkably reduced the phosphorylated level of PI3K, Akt and mTOR (Fig. 6e) without affecting their total protein levels, indicating the inhibition on PI3K/Akt/mTOR pathway. Moreover, NADA also inhibited the phosphorylation of Erk and STAT3. The inhibitory effect on these signaling molecules sensitized the cytotoxicity of cisplatin against HeLa cells and the combined use of NADA and cisplatin showed clear synergistic effect (Fig. 6f). We therefore concluded that NADA degraded HPV E6/E7 and consequently depressed their signaling functions.

### UPS-dependent pathway involves in NADA-induced E6/E7 degradation

**Figure 7:**
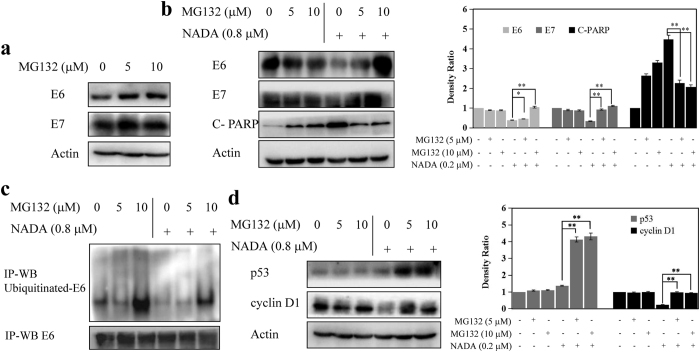
NADA induced E6/E7 viral oncoproteins degradation via UPS pathway. (**a**) E6/E7 were degraded by the proteasome in HeLa cells. HeLa cells were treated with 5 and 10 μM MG132 for 6 h, and then the expression levels of E6/E7 were examined by western blotting. (**b**) NADA induced E6/E7 degradation was mediated by proteasome. HeLa cells were treated with 5 or 10 μM MG132 for 1 h before the addition of 0.8 μM NADA for another 2 h, and then the expression levels of E6/E7 and C-PARP were examined by western blotting; the intensity of protein bands using gray analysis are shown in histograms. (**c**) NADA-induced degradation was mediated by UPS. HeLa cells were pretreated with 5 or 10 μM MG132 for 1 h and then treated with 0.8 μM NADA for another 2 h. Whole cell lysates were subjected to IP with anti-E6 antibodies, and then detected by western blotting with anti-ubiquitin or anti-E6 antibodies. (**d**) Enhanced proteasome activity resulted degradation of p53 and cyclin D1; the intensity of protein bands using gray analysis are shown in histograms. Values represent the means ± SD. *P < 0.05, **P < 0.01 versus control. All experiments were performed in three replicates (n = 3).

Under normal conditions, E6/E7 usually degrade via the UPS^9^. In our experiments, we found that MG132 (a proteasome inhibitor) led to increased E6/E7 (Fig. 7a), confirming E6/E7 were degraded by proteasome in HeLa cells. In order to explore whether proteasome is also responsible for NADA-induced E6/E7 degradation, HeLa cells were pre-incubated with MG132 before NADA treatment. As shown in Fig. 7b, NADA alone induced obvious decrease of E6/E7. However, in the presence of MG132, the expression levels of E6/E7 were recovered, indicating NADA-induced E6/E7 degradation was mediated by proteasome, and NADA had an opposite function against MG132. C-PARP was decreased after co-treated with MG132 and NADA, indicating E6/E7 degradation was necessary for NADA-induced apoptosis in HeLa cells and recover of E6/E7 rescued cells from NADA-induced cell death (Fig. 7b). Ubiquitination of proteins are needed when degraded by proteasome^37^,^38^. Our results showed that NADA could reduce the ubiquitination of E6. However, when pretreated with MG132, the ubiquitinated E6 accumulated remarkably (Fig. 7c), suggesting NADA enhanced the activity of proteasome and subsequently reduced the level of ubiquitinated E6. These results (Fig. 7b,c) demonstrated that NADA induced E6/E7 degradation by stimulating UPS. As mentioned above (Fig. 6e), the expression level of p53 and cyclin D1, which are two substrates of the UPS, decreased after treatment with NADA. Our further experiments showed that NADA-induced decrease in the level of p53 and cyclin D1 attenuated significantly in the presence of a proteasome inhibitor (Fig. 7d), which indicated that the stimulated UPS was account for the decrease of p53 and cyclin D1. Therefore, we concluded that enhanced UPS activity was the cause of NADA-triggered E6/E7 degradation.

### NADA-induced reduction in E6/E7 is dependent on ROS accumulation and ROS play a role in the upstream of proteasome

**Figure 8:**
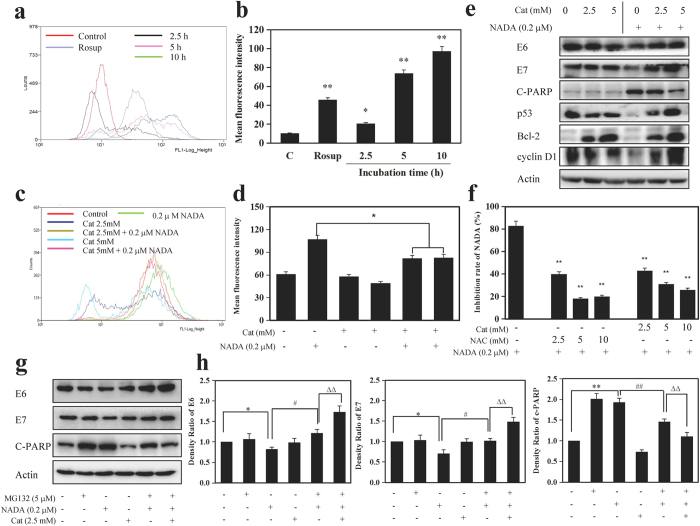
NADA-induced ROS accumulation was involved in E6/E7 degradation, HeLa cell apoptosis, and enhanced UPS activity. (**a**) NADA increases ROS generation in HeLa cells. HeLa cells were treated with 0.2 μM NADA for indicated times, and intracellular ROS levels were detected. (**b**) Histogram shows the increase of the DCF fluorescence after treatment with NADA. Values represent the means ± SD. *P < 0.05, **P < 0.01 versus control. (**c**) HeLa cells were pretreated with, or without, 2.5 or 5 mM Cat for 1 h followed by 0.2 μM NADA for 6 h, and ROS levels were detected. (**d**) Histogram shows intensity of the DCF fluorescence. (**e**) HeLa cells were pretreated, or not, for 1 h with 2.5 or 5 mM Cat followed by 0.2 μM NADA for 6 h, the indicated proteins were assessed by western blotting, and the histograms showing the quantity analysis for intensity of the bands are shown as Supplementaray Fig. S3. (**f**) Antioxidants Cat and NAC attenuated the NADA-induced cytotoxicity. HeLa cells were pretreated with, or without, NAC or Cat (2.5–10 mM) for 1 h, and then treated with 0.2 μM NADA for 24 h, and cell viability was assayed by MTT method. (**g**) HeLa cells were pre-incubated with or without 5 μM MG132 for 1 h and then exposed to Cat (2.5 mM) for 1 h, then NADA (0.2 μM) for 4 h, or a combination of both. Where indicated, Cat was added 1 h before NADA treatment, and the indicated proteins were analyzed by western blotting. (**h**) Histograms show the intensity of protein bands use gray analysis method. Values represent the means ± SD. *P < 0.05, **P < 0.01 versus control; ^∆^*P* < 0.05, ^∆∆^*P* < 0.01 versus the group of NADA; ^#^*P* < 0.05, ^##^*P* < 0.01 versus the groups of MG132 and NADA. All experiments were performed in three replicates (n = 3).

It has been shown that ROS has a close relationship with the activity of UPS^39^ and cell apoptosis. Therefore, we examined whether NADA could stimulate the generation of ROS. After exposure to NADA for 2.5, 5, 10 h, the intensity of dichlorodihydrofluorescein (DCF) fluorescence increased in a time-dependent manner in HeLa cells (Fig. 8a,b), indicating NADA-induced increasing of ROS generation. Such an increase in the generation of ROS can be attenuated by the ROS scavenger catalase (Cat, a natural antioxidant), as shown in Fig. 8c,d. To further investigate whether NADA-induced apoptosis and reduction in E6/E7 was ROS dependent, we pretreated HeLa cells with Cat. Our results showed that NADA-induced degradation of E6/E7 restored in the presence of Cat, suggesting increased ROS generation contributed to the decrease of E6/E7. Moreover, ROS scavenger Cat could reduce the production of C-PARP, and thereof prevented NADA triggered apoptosis in HeLa cells (Fig. 8e), indicating recovered E6/E7 rescued cells from NADA-induced cell death and ROS was indispensable to the apoptosis stimulated by NADA. Furthermore, pre-incubation with Cat or other antioxidant NAC also inhibited NADA-mediated inhibition on cell proliferation (Fig. 8f), further confirming ROS was necessary for NADA-induced cell death. These results suggested that NADA can enhance ROS production, which regulated the degradation of E6/E7 and HeLa apoptosis. ROS has been previously reported to be implicated in the regulation of UPS components^14^. In our present investigation, UPS substrates p53, Bcl-2, and cyclin D1, which decreased after NADA treatments, also recovered after ROS scavenge (Fig. 8e), implying an indirect effect of NADA on UPS, and possible ROS functions in the upstream of UPS. It is thus possible that ROS may be the cause of NADA-induced UPS activation and subsequent E6/E7 degradation. To test this hypothesis, HeLa cells were untreated or pre-incubated with MG132, and then treated with Cat, NADA, or a combination of Cat and NADA, and finally, the levels of E6/E7 and C-PARP were measured. As already shown in Fig. 7b, NADA-induced degradation of E6/E7 can recover by MG132. Interestingly, in the presence of ROS scavenger Cat, E6/E7 was increased, and C-PARP was decreased, compared to the combined treatment with MG132 and NADA (Fig. 8g,h). These results suggested ROS functioned in the upstream of UPS, and induced the enhanced proteasome function and E6/E7 degradation.

## Discussion

Antimycin-type compounds are a family of natural products that share a common structural skeleton consisting of a macrocyclic ring with an amide linkage to a 3-formamidosalicylate unit^40^. The most well-known of these compounds is AMA. AMA is produced by *Streptomyces* sp. and an inhibitor of mitochondrial electron transport chain that induces extra ROS in biological systems^41^,^42^. AMA can induce ROS accumulation and mitochondrion-dependent apoptosis in HeLa^43^,^44^ and PC12 cells^45^. In our present work, we also discovered the inhibition on the mitochondrial respiration and disruption on the MMP in the presence of NADA (Fig. 5). Collectively, it seems that the inhibition on the mitochondrial respiration is the common mechanisms for the cytotoxicity of these AMA compounds. AMA is also an inhibitor of Bcl-2 and AMA analogues dock well to antiapoptotic protein Bcl-2 and inhibit its antiapoptotic function^46^,^47^. 2-methoxy AMA, an AMA analogue, binds and inhibits Bcl-2 family member Bcl-xl and destroys the mitochondrion function^48^. Results from preclinical pharmacology studies suggested 2-methoxy AMA is a good candidate for further clinical development^49^. Much attention has been paid to the structural searching, biosynthesis, chemical synthesis, and bioactivities of AMA analogues to develop a novel family of anticancer compounds^40^. In this present work, we have isolated and characterized several AMA analogues, including four new and six known ones, from a marine derived *Streptomyces* sp. THS-55. All these compounds showed cytotoxicity against HeLa cells, and the primary structure-activity relationship showed that the acyl group linked to 3′-NH seems to be essential to the cytotoxicity. Nonetheless, this hypothesis needs additional experimental evidences such as deformylation of these compounds and then reevaluation of their activities. The most active compound NADA possessed proliferation-inhibitory and proapoptotic effects in HeLa cells. Our results further revealed that HPV-positive cancer cells were more sensitive to NADA than the other HPV-negative cell lines (Fig. 3b); furthermore, silence of HPV oncoprotein E6 by siRNA decreased the sensitivity of HPV-positive cells to NADA (Supplementary Fig. S4), suggesting its selectivity to the HPV-positive cancer cells.

The oncoproteins E6/E7 contribute to the malignant phenotype of HPV-infected cervical cancers and regulates many molecules that function in cell survival and apoptosis^50^. In this study, for the first time, we reported that NADA downregulated E6/E7 levels and induced apoptosis in HPV E6/E7 harboring HeLa cells. Restoration of p53/Rb has been proved to be a vital mechanism underlying the E6/E7 degradation-induced apoptosis^51^. Our mechanistic investigation showed that NADA-induced degradation of E6/E7 and stimulation of apoptosis were not completely associated with the restoration of p53/Rb, because NADA could only recover the expression level of Rb, but decrease the level of p53. This phenomenon was different from other compounds, *e.g.* anisomelic acid, which recover the expression level of p53 when E6/E7 degrade^18^. The decreased expression level of p53 after NADA treatment also revealed that NADA-induced apoptosis was p53 independent. P53 mutate or null in about half of the human tumors and functionally defective p53 reduces sensitivity to anticancer agents, therefore, the competence of NADA inducing HeLa apoptosis independently of p53 provides a therapeutic advantage for the treatment of p53 defective tumors. NADA-induced apoptosis was p53-independent but caspase-dependent, because a pan caspase inhibitor Z-VAD-FMK could reverse NADA-induced apoptosis. After NADA treatment, Rb increased, however, the HeLa cell cycle was arrested at S phase rather than G1 phase. This is the same as AMA^44^ (a mitochondria damage agent) and Tanshinone IIA^17^(an E6/E7 decreasing compound), both induced S phase arrest. Possibly, E6/E7 degradation and the restoration of Rb did not play a definitive role in regulating NADA-induced cell S arrest. Apart from the Rb restoration, PI3K/Akt/mTOR pathway, Erk1/2, and STAT3 were inhibited by NADA. Because many signaling pathways or molecules are activated by E6/E7, multiple signaling molecules need to be repressed simultaneously for the treatment of cervical cancers. Therefore, E6/E7 degradation and modulation of E6/E7 downstream molecules were responsible for the NADA-induced apoptotic cell death. Meanwhile, NADA also showed cytotoxicity to HPV-negative cancer cells. It is possible that inhibition of mitochondrial respiration, inhibition of anti-apoptotic proteins Bcl-2/Bcl-xL as well as modulation of specific cancer related molecules^40^, are all responsible for the action of NADA in certain cells.

In our experiments to explore the reason for E6/E7 degradation, we found that E6/E7 degradation had a relationship with the NADA-induced UPS activity enhancement. Inhibition of UPS function with MG132 alleviated NADA-induced degradation of E6/E7, indicating that the increased activation of UPS involved in the NADA-induced degradation of E6/E7. Moreover, we found the MG132 inhibition of UPS function also recovered the level of two other endogenous UPS substrates, p53 and cyclin D1, conforming the activation of UPS by NADA treatment.

Our results also showed NADA disrupted MMP and increased ROS accumulation in HeLa cells, which was mainly caused by the mitochondrial respiration inhibition. We compared the effect on mitochondrial respiration, ROS generation, and cytotoxicity of compound **1**, **2**, **3**, **6**, **8**, **10**, and NADA. We found that, the generation of ROS had a positive correlation with their mitochondrial respiration inhibition (Supplementary Fig. S5) and cytotoxicity, supporting the causative role of mitochondrial respiration inhibition and ROS generation in NADA-induced cytotoxicity. NADA-induced ROS repressed E6/E7 levels, and this function could be attenuated by an antioxidant agent, suggesting that the NADA-induced increasing function of UPS was not via the direct effect of NADA on UPS, but via an indirect effect of ROS. Furthermore, we confirmed that UPS activation mediated by upstream ROS was account for the NADA-induced E6/E7 loss, evidenced by the fact that, ROS scavenger Cat could reverse the increased UPS activity that were stimulated by NADA. Another finding in our work was that ROS scavenger could reverse the NADA-induced decrease in Bcl-2. Other researchers also reported that ROS could induce the degradation of Bcl-2^52^. Thus, we concluded that NADA-induced decreases in the Bcl-2 level was also the result of ROS accumulation, which may also explain the decreased Bcl-2 level induced by AMA in other studies^43^, and provided another mechanism to regulate Bcl-2 in addition to direct interaction with Bcl-2^44^.

ROS can enhance ubiquitination^53^ and lead to the increased accumulation of Ub-conjugates, which enhances the activity of UPS^39^. Therefore, formation of Ub-conjugates was a necessary process for the UPS degradation. However, after NADA treatment, we observed a decreased rather than increased level of Ub-E6. Under the same conditions, UPS inhibitor MG132 increased the level of Ub-E6, showing contrary effects on ubiquitination to NADA. A possible explanation for such results is that under the present experimental conditions, in the presence of NADA, more Ub-E6 was degraded by the increased activity of UPS, while in the presence of MG132, more Ub-E6 was accumulated when the activity of UPS was inhibited.

AMA and its analogue are widely distributed in *Streptomyces* sp. and exhibit a wide range of biological activities, including antifungal^54^, insecticidal^55^, and nematocidal activities^56^. Our present studies revealed that this type of compounds, such as NADA, also possessed potent cytotoxicity to HPV infected cancer cells with a unique underlying mechanism that decreases oncoptroteins E6/E7. Other NADA analogues, for example, compound **4**, also decrease HeLa oncoptroteins E6/E7 (Supplementary Fig. S6), and more detailed work are under way in our laboratory. These results indicated that these compounds could be developed as a novel class of cervical cancer therapeutic agents.

In summary, our present study demonstrated that marine-derived AMA analogue NADA induced apoptotic death in E6/E7 positive cells and a unique mechanism of E6/E7 degradation via stimulating the ROS-mediated UPS function. NADA simultaneously activated UPS function and degraded E6/E7 in cervical cancer cells, which highlighted a novel pharmaceutical function of this type of compounds, and revealed a novel mechanism of NADA-stimulated HPV-associated cancer cells death. The present AMA analogues could serve as lead compounds for anticancer agents as well as research tools to dissect E6/E7 functions.

## Methods

### General

IR spectra were recorded on a Nicolet NEXUS 470 spectrophotometer in KBr discs. CD spectra were measured on JASCO J-715. UV spectra were recorded on Beckman DU640 spectrophotometer. Optical rotations were obtained on a JASCO P-1020 digital polarimeter. NMR spectra were recorded on an Agilent NMR spectrometer using CDCl_3_ as solvent and tetramethylsilane (TMS) as an internal standard. ESIMS was measured on a Micromass Q-TOF Ultima Global GAA076 LC mass spectrometer. HRESIMS was measured on a Micromass EI-4000 (Autospec-Ultima-TOF). Semi-preparative HPLC was performed using an ODS column (YMC-Pack ODS-A, 5 μm, 10 × 250 mm, 3 mL/min). Racemic mixtures were resolved on Chiralpak IA and Chiralpak IB columns (5 μm, 4.6 × 250 mm, hexane-isopropanol eluent, and 1 mL/min). Column chromatography (CC) was performed on silica gel (200–300 mesh) and Sephadex LH-20.

### Actinomycetes material

The strain THS-55 was isolated from the sediment of mangrove, Sanya, China. It was classified as a *Streptomyces* sp. based on 16S rDNA analysis (Genbank No. KM103736).

### Fermentation and extraction

The strain THS-55 was cultivated in an Erlenmeyer flask (500 mL) containing 150 mL of a defined medium with the following components: 10 g glucose, 10 g soluble starch, 5 g peptone, 3 g yeast extract, 2 g beef extract, 2 g soy flour, 0.05 g K_2_HPO_4_, 0.05 g MgSO_4_·7H_2_O, 0.2 g KBr, 2 g CaCO_3_, dissolved in 1 liter of seawater, pH 7.0. Each flask was inoculated spores of the organism and incubated on a rotary shaker (180 r.p.m.) at 28 °C for 7 days. After cultivation, 80 L of whole broth was extracted three times with EtOAc. The organic extract was concentrated to dryness in vacuo to afford crude material (25.0 g).

### Purification

The extract (25.0 g) was applied to a silica gel column chromatography and was separated into six fractions (Fr.1-Fr.6) using a step gradient elution of petroleum ether-acetone and CH_2_Cl_2_-CH_3_OH. Fr.3, eluted with CH_2_Cl_2_ and 100:1 CH_2_Cl_2_-MeOH, was fractionated on a C-18 ODS column using a step gradient elution of MeOH/H_2_O and was separated into 6 subfractions (Fr.3.1-Fr.3.6). Fr.3.4 was further purified by Sephadex LH-20 (MeOH) and purified by semipreparative HPLC (80:20 MeOH/H_2_O, 3 mL/min) to yield the compound compound **6** (20 mg; *t*_R_ 20 min), Fr.3.4.1 and Fr.3.4.2. Fr.3.4.1 and Fr.3.4.2. were further purified by using a chiral-phase HPLC column respectively (85:15 hexane/isopropanol, 1 mL/min) to yield the compound **4** (1 mg; *t*_R_ 20 min), compound **5** (3 mg; *t*_R_ 21 min), and compound **7** (3 mg; *t*_R_ 18 min). Fr.3.3 was also purified by as further purified by Sephadex LH-20 (MeOH) and purified by semipreparative HPLC (80:20 MeOH/H_2_O, 3 mL/min) to yield the compound **1** (15 mg; *t*_R_ 12 min), compound **8** (10 mg; *t*_R_ 7 min), compound **9** (3 mg; *t*_R_ 8.5 min), compound **10** (21 mg; *t*_R_ 12.5 min) and Fr.3.3.1. Fr.3.3.1 was further purified by using a chiral-phase HPLC column (90:10 hexane/isopropanol, 1 mL/min) to yield the compound **2** (3 mg; *t*_R_ 15 min), compound **3** (1 mg; *t*_R_ 17 min).

**Antimycin E (1):** White powder; CD (0.81 × 10^−3^ M, MeOH) *λ*_max_ (Δ*ε*) 231.5 (+0.90) nm; UV (MeOH) *λ*_max_ (log *ε*) 231 (2.74), 316 (0.60) nm; [α]^20^_D_ = +43.6 (*c* 2.0, MeOH); IR (KBr) *ν*_max_ 3361, 2937, 1733, 1541, 1373, 1233, 1201, 754 cm^−1^; ^1^H and ^13^C NMR spectral data, see Supplementary Tables S1 and S2; HRESIMS *m/z* 493.2189 [M + H]^+^ (calcd for C_24_H_33_N_2_O_9_, 493.2181).

**Antimycin F** **(2):** White powder; CD (0.77 × 10^−3^ M, MeOH) *λ*_max_ (Δ*ε*) 230.5 (+0.96) nm; UV (MeOH) *λ*_max_ (log *ε*) 231 (2.95), 316 (0.71) nm; [α]^20^_D_ = +34.8 (*c* 2.0, MeOH); IR (KBr) *ν*_max_ 3291, 2958, 1748, 1540, 1373, 1182, 1145, 749 cm^−1^; ^1^H and ^13^C NMR spectral data, see Supplementary Tables S1 and S2; HRESIMS *m/z* 521.2487 [M + H]^+^ (calcd for C_26_H_37_N_2_O_9_, 521.2494).

**Antimycin G (3):** White powder; CD (0.77 × 10^−3^ M, MeOH) *λ*_max_ (Δ*ε*) 230.5 (+1.12) nm; UV (MeOH) *λ*_max_ (log *ε*) 231 (2.95), 316 (0.71) nm; [α]^20^_D_ = +33.5 (*c* 2.0, MeOH); IR (KBr) *ν*_max_ 3291, 2958, 1748, 1540, 1373, 1182, 1145, 749 cm^−1^; ^1^H and ^13^C NMR spectral data, see Supplementary Tables S1 and S2; HRESIMS *m/z* 521.2482 [M + H]^+^ (calcd for C_26_H_37_N_2_O_9_, 521.2494).

**Antimycin H (4):** White powder; CD (0.89 × 10^−3^ M, MeOH) *λ*_max_ (Δ*ε*) 234.0 (+1.37) nm; UV (MeOH) *λ*_max_ (log *ε*) 233 (2.81), 316 (0.64) nm; [α]^20^_D_ = +46.2 (*c* 2.0, MeOH); IR (KBr) *ν*_max_ 3356, 2930, 1748, 1540, 1374, 1178, 1144, 753 cm^−1^; ^1^H and ^13^C NMR spectral data, see Supplementary Tables S1 and S2; HRESIMS *m/z* 563.2947 [M + H]^+^ (calcd for C_29_H_43_N_2_O_9_, 563.2963).

**N-acetyl-deformylantimycin A (5):** White powder; CD (0.89 × 10^−3^ M, MeOH) *λ*_max_ (Δ*ε*) 234.0 (+1.27) nm; UV (MeOH) *λ*_max_ (log *ε*) 233 (2.81), 316 (0.64) nm; [α]^20^_D_ = +42.2 (*c* 2.0, MeOH); IR (KBr) *ν*_max_ 3356, 2930, 1748, 1540, 1374, 1178, 1144, 753 cm^−1^; ^1^H and ^13^C NMR spectral data, see Supplementary Tables S1 and S2; HRESIMS *m/z* 563.2950 [M + H]^+^ (calcd for C_29_H_43_N_2_O_9_, 563.2963). The raw NMR charts of these new compounds **1**–**5** are presented in Supplementary Figs S7–36.

**Deformylated antimycin A**_**2a**_ **(6):** White powder; UV (MeOH) *λ*_max_ (log *ε*) 231 (2.68), 316 (0.62) nm; [α]^30^_D_ = +17.7 (*c* 2.0, MeOH); IR (KBr) *ν*_max_ 3385, 2931, 1748, 1540, 1457, 1184, 1144, 741 cm^−1^; ^1^H and ^13^C NMR spectral data, see Supplementary Tables S1 and S2; HRESIMS 507.2716 [M + H]^+^ (calcd for C_26_H_39_N_2_O_8_, 507.2701).

**Deformylated antimycin A**_**1a**_ **(7):** White powder; UV (MeOH) *λ*_max_ (log *ε*) 231 (2.84), 316 (0.67) nm; [α]^30^_D_ = +19.6 (*c* 2.0, MeOH); IR (KBr) *ν*_max_ 3566, 2925, 1748, 1541, 1457, 1186, 1143 cm^−1^; ^1^H NMR spectral data, see Supplementary Table S2; ESIMS *m*/*z* 521.3 [M + H]^+^.

**Antimycin A**_**18**_ **(8):** White powder; UV (MeOH) *λ*_max_ (log *ε*) 231 (2.72), 316 (0.63) nm; [α]^30^_D_ = +34.0 (*c* 2.0, MeOH); IR (KBr) *ν*_max_ 3545, 2958, 1749, 1540, 1457, 1184, 1038 cm^−1^; ESIMS *m/z* 479.3 [M + H]^+^.

**Antimycin A**_**6a**_**(9):** White powder; UV (MeOH) *λ*_max_ (log *ε*) 231 (2.85), 316 (0.72) nm; [α]^30^_D_ = +28.0 (*c* 2.0, MeOH); IR (KBr) *ν*_max_ 3366, 2933, 1748, 1540, 1457, 1362, 1185, 1144, 749 cm^−1^; ESIMS *m*/*z* 479.1 [M + H]^+^.

**Antimycin A**_**4a**_**(10):** White powder; UV (MeOH) *λ*_max_ (log *ε*) 231 (2.96), 316 (0.66) nm; [α]^30^_D_ = +32.0 (*c* 2.0, MeOH); IR (KBr) *ν*_max_ 3366, 2933, 1748, 1540, 1557, 1362, 1181, 1144, 747 cm^−1^; ESIMS *m*/*z* 507.4 [M + H]^+^.

### Cell viability assessment and calculation of drugs synergism

Cell viability were assayed by 3-(4, 5-dimethylthiazol-2-yl)-2, 5-diphenyltetrazolium bromide (MTT) methods^57^. The synergistic effect of multiple drugs was calculated by the definition of Chou and Talalay^58^.

### Colony formation assay

HeLa cells were plated onto 6-well plates (800 cells/well). After 24 h, NADA (0–2.56 μM) was added and incubated for 12 days. Cells were washed, fixed in methanol, and stained with Giemsa. Finally, colonies were scored and photographed.

### Measurement of MMP

HeLa cells were seeded in six-well plates and incubated with NADA (0–0.1 μM) for 24 h, and then stained with Rho 123 (3 μg/mL), and the MMP were analyzed^59^.

### Mitochondrial respiration assay

HeLa cells were seeded 40,000/well. After 24 h, the inhibition on the mitochondrial respiration was assayed using the extracellular O_2_ consumption assay kit (Abcam, Cambridge, USA), according to the manufacture’s protocols.

### Cell cycle analysis

HeLa cells were incubated with NADA (0–0.1 μM) for 24 h. After that, the cells were collected and washed in PBS and fixed in ice-cold 70% (v/v) ethanol overnight at −20 °C. The samples were prepared and analyzed as described previously^59^.

### Hoechst 33342 staining assay

HeLa cells were treated with NADA (0–0.1 μM) of for 24 h, the cells were washed twice with PBS and stained with Hoechst 33342 (5 μg/mL) for 30 min at 4 °C in the dark. Finally, the nuclei of apoptotic cells were detected by fluorescence microscopy.

### Western blotting and immunoprecipitation

For immunoprecipitation, after treatment, HeLa cells were lysed. The supernatants were incubated with HPV-18 E6 antibody and protein A-Sepharose beads at 4 °C overnight. Subsequently, beads were washed and boiled in loading buffer for 12 min, and then, proteins were separated by 15% SDS-PAGE gels for ubiquitination analysis and detection of E6 protein with western blotting analyses^59^. Information for the antibodies is shown in Supplementary Table S5.

### Measurement of ROS generation

HeLa cells were treated with 0.2 μM NADA for 2.5, 5, and 10 h, respectively. In another way, cells were pretreated, or not, with 2.5 mM, 5 mM Cat for 1 h followed by 0.2 μM NADA for 6 h. Then, cells were incubated with CM-H2DCFDA probes for 20 min and analyzed by flow cytometry.

### Data analysis

One-way ANOVA with Tukey’s post hoc test was implemented for all statistical analysis, and values were expressed as mean ± SD. Differences of *P* < 0.05 or *P* < 0.01 were considered statistically significant.

## Additional Information

**How to cite this article**: Zhang, W. *et al*. Marine *Streptomyces* sp. derived antimycin analogues suppress HeLa cells via depletion HPV E6/E7 mediated by ROS-dependent ubiquitin-proteasome system. *Sci. Rep.* **7**, 42180; doi: 10.1038/srep42180 (2017).

**Publisher's note:** Springer Nature remains neutral with regard to jurisdictional claims in published maps and institutional affiliations.

## Supplementary Material

Supplementary Information
