# Supplementary material for: Marine Streptomyces sp. derived antimycin analogues suppress HeLa cells via depletion HPV E6/E7 mediated by ROS-dependent ubiquitin–proteasome system
Source: Sci Rep. 2017 Feb 8;7:42180. doi: 10.1038/srep42180 (PMC5296914; doi:10.1038/srep42180)
Supplement: Supplementary Information [file srep42180-s1.pdf]

Marine *Streptomyces* sp. derived antimycin analogues suppress HeLa cells via depletion HPV E6/E7 mediated by ROS-dependent ubiquitin–proteasome system

Weiyi Zhang[1, +], Qian Che[1, 2, +], Hongsheng Tan[1], Xin Qi[1, 2], Jing Li[1, 2], Dehai Li[1, 2], Qianqun Gu[1, 2], Tianjiao Zhu[1, 2, *], and Ming Liu[1, 2, *]

SI Tables

Table S1. ^{13}C NMR data for compounds 1-5 (recorded in $CDCl_3$)

	**1**[a]	**2**[a]	**3**[a]	**4**[a]	**5**[a]
position	δ_C, type	δ_C, type	δ_C, type	δ_C, type	δ_C, type
2	170.0, C	170.1, C	170.1, C	170.1, C	170.1, C
3	53.7, CH	53.6, CH	53.6, CH	53.7, CH	53.7, CH
4	70.9, CH	70.9, CH	70.9, CH	70.9, CH	70.9, CH
6	172.9, C	172.9, C	172.9, C	173.0, C	173.0, C
7	50.2, CH	50.1, CH	50.1, CH	50.1, CH	50.1, CH
8	75.7, CH	75.2, CH	75.2, CH	75.3, CH	75.3, CH
9	74.7, CH	74.8, CH	74.8, CH	74.8, CH	74.8, CH
10	17.8, CH_3	17.8, CH_3	17.8, CH_3	17.9, CH_3	17.9, CH_3
11	15.0, CH_3	15.0, CH_3	15.0, CH_3	14.9, CH_3	14.9, CH_3
1''	28.2, CH_2	28.0, CH_2	28.3, CH_2	28.5, CH_2	28.5, CH_2
2''	29.2, CH_2	29.2, CH_2	22.4, CH_2	22.5, CH_2	22.5, CH_2
3''	22.4, CH_2	22.4, CH_2	27.0, CH_2	27.0, CH_2	27.0, CH_2
4''	13.9, CH_3	13.8, CH_3	31.4, CH_2	31.6, CH_2	31.6, CH_2
5''			28.9, CH_2	28.9, CH_2	28.9, CH_2
6''			14.0, CH_3	14.0, CH_3	14.0, CH_3
1'	112.4, C	112.6, C	112.6, C	112.5, C	112.5, C
2'	150.5, C	150.5, C	150.5, C	150.5, C	150.5, C
3'	128.3, C	128.0, C	128.0, C	128.3, C	128.3, C
4'	124.1, CH	124.4, CH	124.4, CH	124.1, CH	124.1, CH
5'	118.9, CH	119.0, CH	119.0, CH	119.0, CH	119.0, CH
6'	119.6, CH	120.0, CH	120.0, CH	119.7, CH	119.7, CH
7'	169.5, C	169.3, C	169.3, C	169.4, C	169.4, C
8'	168.7, C	168.4, C	168.4, C	168.7, C	168.7, C
9'	24.9, CH_3	24.7, CH_3	24.7, CH_3	24.9, CH_3	24.9, CH_3
1'''	169.7, C	175.6, C	169.6, C	175.3, C	175.3, C
2'''	20.8, CH_3	34.1, CH	20.8, CH_3	41.3, CH	43.2, CH_2
3'''		18.9, CH_3		26.5, CH_2	25.5, CH
4'''		18.9, CH_3		11.7, CH_3	22.5, CH_3
5'''				16.8, CH_3	22.5, CH_3

[a] Spectra were recorded at 125 MHz for ^{13}C NMR using TMS as internal standard.

Table S2. ^{1}H NMR data for compounds 1-5 (recorded in $CDCl_3$)

	1[a]	2[a]	3[a]	4[a]	5[a]
position	δ_H (*J* in Hz)	δ_H (*J* in Hz)	δ_H (*J* in Hz)	δ_H (*J* in Hz)	δ_H (*J* in Hz)
3	5.27, dd (5.90,7.50)	5.28, dd (7.35, 7.60)	5.28, dd (7.40, 7.65)	5.28, dd (7.30, 7.65)	5.28, dd (7.40, 7.60)
4	5.72, dq (6.80, 5.90)	5.72,dq (6.80, 7.35)	5.72, dq (6.75, 7.40)	5.72, dq (6.80, 7.30)	5.72, dq (6.85, 7.40)
7	2.49, m	2.53, dt (10.55, 2.70)	2.52, m	2.53, dt (10.55, 2.70)	2.51, dt (10.55, 2.70)
8	5.05, dd (9.80, 9.95)	5.07, dd (9.85, 10.55)	5.07, dd (9.85, 10.55)	5.08, dd (9.85, 10.55)	5.08, dd (9.85, 10.55)
9	4.96, m	4.98, m	4.98, m	4.98, m	4.98, m
10	1.30[b]	1.30[b]	1.30[b]	1.30[b]	1.30[b]
11	1.32[b]	1.32[b]	1.32[b]	1.31[b]	1.31[b]
1''	1.41, m; 1.70, m	1.22, m; 1. 70, m	1.22, m; 1.70, m	1.22, m; 1.70, m	1.22, m; 1.70, m
2''	1.25, m	1.25, m	1.25, m	1.24, m	1.24, m
3''	1.25, m	1.25, m	1.25, m	1.24, m	1.24, m
4''	0.85, t (6.90)	0.87, t (6.90)	1.25, m	1.24, m	1.24, m
5''			1.25, m	1.24, m	1.24, m
6''			0.87, t (6.60)	0.86, t (7.10)	0.87, t (6.80)
4'	8.51, d (7.90)	8.52, d (8.00)	8.52, d (8.00)	8.52, d (8.00)	8.53, d (8.00)
5'	6.90, t (8.00)	6.91, t (8.05)	6.91, t (8.05)	6.91, t (8.10)	6.91, t (8.10)
6'	7.21, d (7.90)	7.21, d (8.00)	7.21, d (8.00)	7.21, d (8.00)	7.21, d (8.00)
9'	2.23, s	2.24, s	2.24, s	2.24, s	2.23, s
NH-3'	7.88, s	7.87, s	7.88, s	7.87, s	7.91, s
NH-3	7.10, d (7.50)	7.08, d (7.60)	7.08, d (7.45)	7.08, d (7.65)	7.08, d (7.60)
OH-2'	12.58, s	12.58, s	12.58, s	12.59, s	12.58, s
2'''	2.12, s	2.61, m	2.13, s	2.42, m	2.24, d (6.40)
3'''		1.20, d (1.70)		1.74, m , 1.50, m	2.13, m
4'''		1.20, d (1.70)		0.95, t (7.40)	0.98, d (6.55)
5'''				1.19, d (7.00)	0.98, d (6.55)

[a] Spectra were recorded at 500 MHz for ^{1}H NMR using TMS as internal standard.

[b] Signals that were overlapped.

Table S3. Cytotoxicity of the selected compounds against different cell lines.

Compounds	IC_{50} (means ±SD, μM)			
	Siha	K562	HL-60	293T
**1**	5.3 ±0.4	11.3 ±0.9	3.7 ±0.2	1.9 ±0.1
**2**	5.6 ±0.5	13.4 ±1.1	5.4 ±0.3	0.8 ±0.0
**3**	7.1 ±0.6	13.8 ±1.2	6.2 ±0.5	3.6 ±0.2
**6**	8.1 ±0.4	7.4 ±0.7	2.9 ±0.2	1.1 ±0.1
**8**	5.4 ±0.6	10.4 ±0.8	0.2 ±0.0	0.9 ±0.0
**10**	4.7 ±0.5	25.6 ±1.5	0.4 ±0.0	0.3 ±0.0

Cells were treated with the compounds for 72 h, and the inhibition rates were assayed by MTT methods.

Table S4. Cytotoxicity for different drugs against three cervical cancer cell lines.

**Compounds**	**IC_{50} (means ± SD, μM)**		
	Siha	CaSki	HeLa
Sorafenib	3.54 ± 0.18	3.95 ± 0.38	1.15 ± 0.16
Imatinib	9.44 ± 0.53	16.37 ± 0.84	9.42 ± 0.42
Cisplatin	4.59 ± 0.27	8.21 ± 0.69	3.84 ± 0.27
Taxol	17.92 ± 0.93	18.75 ± 1.17	0.84 ± 0.13
NADA	0.23 ± 0.01	0.22 ± 0.01	0.02 ± 0.003

Cells were treated with these drugs for 72 h, and the inhibition rates were assayed by MTT methods.

Table S5. Information for antibodies used in the article.

Antibodies	No.	Company
Bax	5023	Cell Signaling Technology (CST)
Bak	6947S	Cell Signaling Technology (CST)
Bcl-2	2870S	Cell Signaling Technology (CST)
Mcl-1	5453S	Cell Signaling Technology (CST)
Bid	2002S	Cell Signaling Technology (CST)
survivin	2808S	Cell Signaling Technology (CST)
C-PARP	5625S	Cell Signaling Technology (CST)
C-cas 9	9505S	Cell Signaling Technology (CST)
C-cas 3	9661S	Cell Signaling Technology (CST)
Cas 3	9662S	Cell Signaling Technology (CST)
Cas 8	9746	Cell Signaling Technology (CST)
HPV-18 E7	GTX40864	GeneTex
HPV-18 E6	GTX20070	GeneTex
HPV-18 E6 (N-17)	sc-1586	SANTA CRUZ
p53	2524	Cell Signaling Technology (CST)
Rb	9309S	Cell Signaling Technology (CST)
cyclin D1	2261	Cell Signaling Technology (CST)
P-PI3k	sc-12929	SANTA CRUZ
PI3k	sc-1637	SANTA CRUZ
P-Erk	9101S	Cell Signaling Technology (CST)
Erk	9102S	Cell Signaling Technology (CST)
P-mTOR	2974S	Cell Signaling Technology (CST)
P-Akt	4060S	Cell Signaling Technology (CST)
Akt	9272S	Cell Signaling Technology (CST)
P-STAT 3	2281-1	EPITOMICS
STAT 3	2236-1	EPITOMICS
β-actin	EM21002	SANTA CRUZ

SI Figures

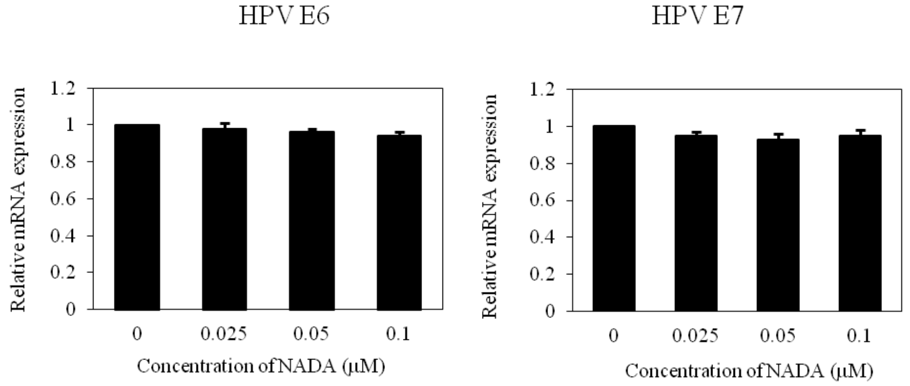

Fig. S1. NADA does not decrease the mRNA level of E6/E7. HeLa cells were treated with NADA (0-0.1 μM) for 24 h and HPV16 E6/E7 mRNA levels were measured by qPCR. qPCR reactions were performed by the ABI7500 system (Applied Biosystems, CA, USA) and SYBR green dye (Roche, Basle, Switzerland). The primer sequences were as follows:

5'-CAGGGCTGCTTTTAACTCTGGT-3'(forward) and

5'-CCTGGAAGATGGTGATGGGAT-3'(reverse) for GAPDH;

5'- CACAGGAGCGACCCAGAAAG-3'(forward) and

5'-GCATAAATCCCGAAAAGCAAAG -3'(reverse) for E6;

5'-CAGAGGAGGAGGATGAAATAGATG-3'(forward) and

5'- GCACAACCGAAGCGTAGAGTC-3'(reverse) for E7.

Data represent means ± SD of relative mRNA expression to the untreated cells.

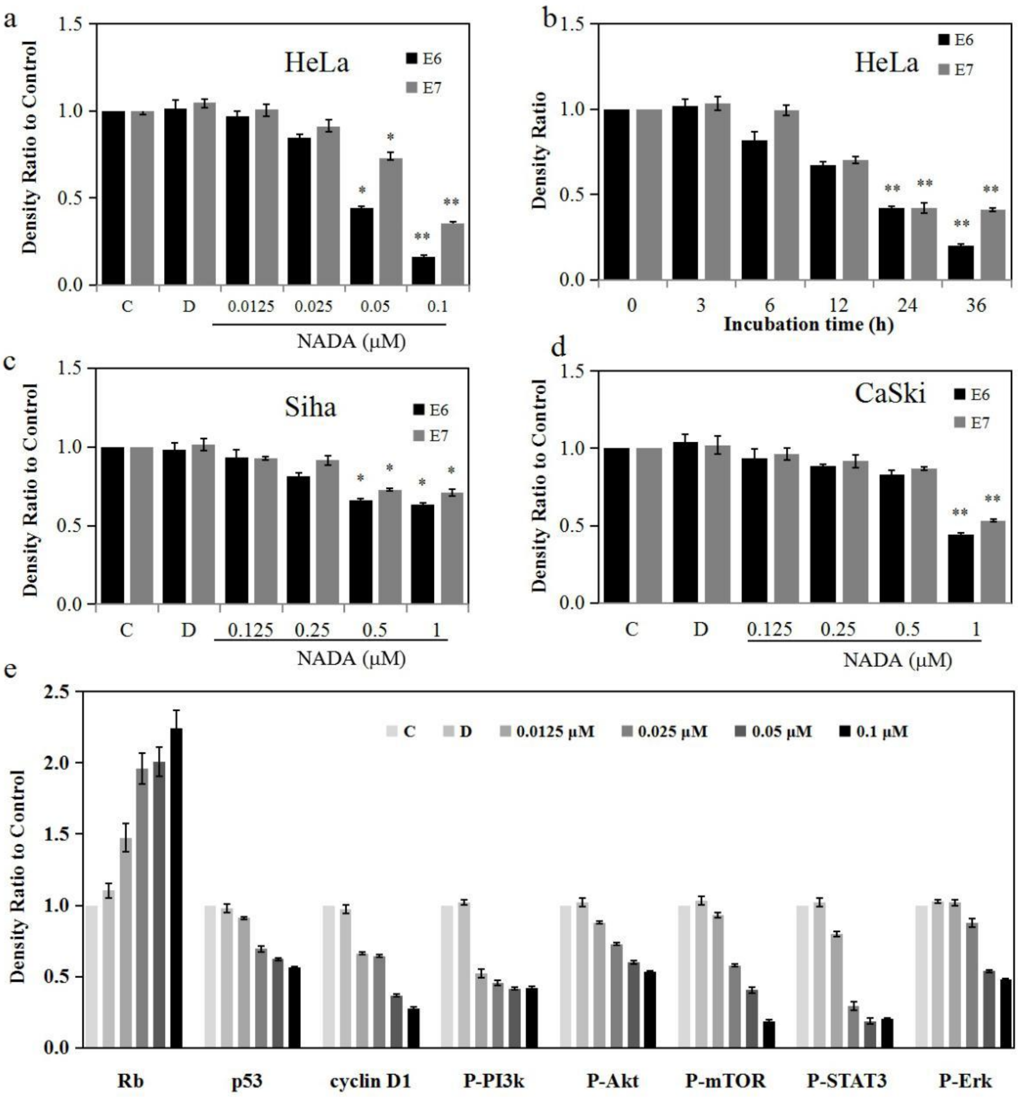

Fig. S2. Histograms show the relative intensity of protein bands in Figure 6 a, b, c, d, and e, using gray analysis method. Values represent the means ± SD. $^{*}P < 0.05$, $^{**}P < 0.01$ versus control group. C, control; D, DMSO.

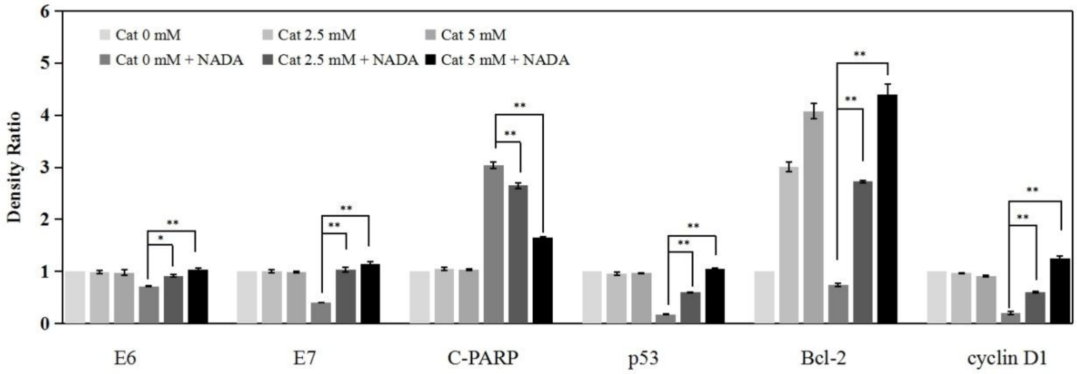

Fig. S3. Histograms show the relative intensity of protein bands in Figure 8e using gray analysis method. Values represent the means ± SD. * $P < 0.05$, ** $P < 0.01$ versus the NADA group.

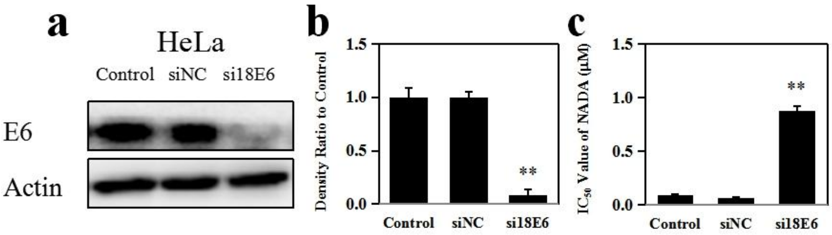

Fig. S4. To confirm the specificity effect on HPV oncoprotiens, we silenced HPV18 E6 in HeLa and examined its sensitivity to NADA. The results showed that HPV-18 E6 siRNA (si18E6) could markedly downregulate E6 expression (a, b). The IC_{50} value of NADA against this E6 low expressing HeLa cells were much larger than the wild-type HeLa cells, indicating HPV oncoprotiens downregulation could reduced the sensitivity of HeLa cells to NADA.

(a) si18E6 downregulates the expression of E6. Cells were seeded into 6-well plates and reached 70% confluency before transfection. siRNAs for HPV18 E6, and scramble siRNA (siNC) were complexed with Lipofectamine 3000 (Invitrogen, Shanghai, China) according to the manufacturer’s instructions and applied to each well, respectively. The transfection medium was removed and replaced with complete medium after 6 h. Then the expression level of E6 was assessed by western blotting after 24 h. siRNAs were synthesized by GenePharma (Shanghai, China) and the sequences are as follows:

si18E6 sense: 5′-CAUUUACCAGCCCGACGAGTT-3′ and

antisense:5′-CUCGUCGGGCUGGUAAAUGTT-3′;

siNC sense:5′-UUCUCCGAACGUGUACGUdTdT-3′ and

antisense: 5′-ACGUGACACGUUCGGAGAAdTdT-3′.

(b) Histogram shows the relative intensity of E6 protein bands using gray analysis method. Values represent the means ± SD. $^{*}P < 0.05$, $^{**}P < 0.01$ versus the NADA group. (c) NADA shows less cytotoxicity against HeLa cells with E6 slienced. Transfected cells were treated with the indicated concentrations of NADA for 48 h, and the cell viability were assayed by MTT method.

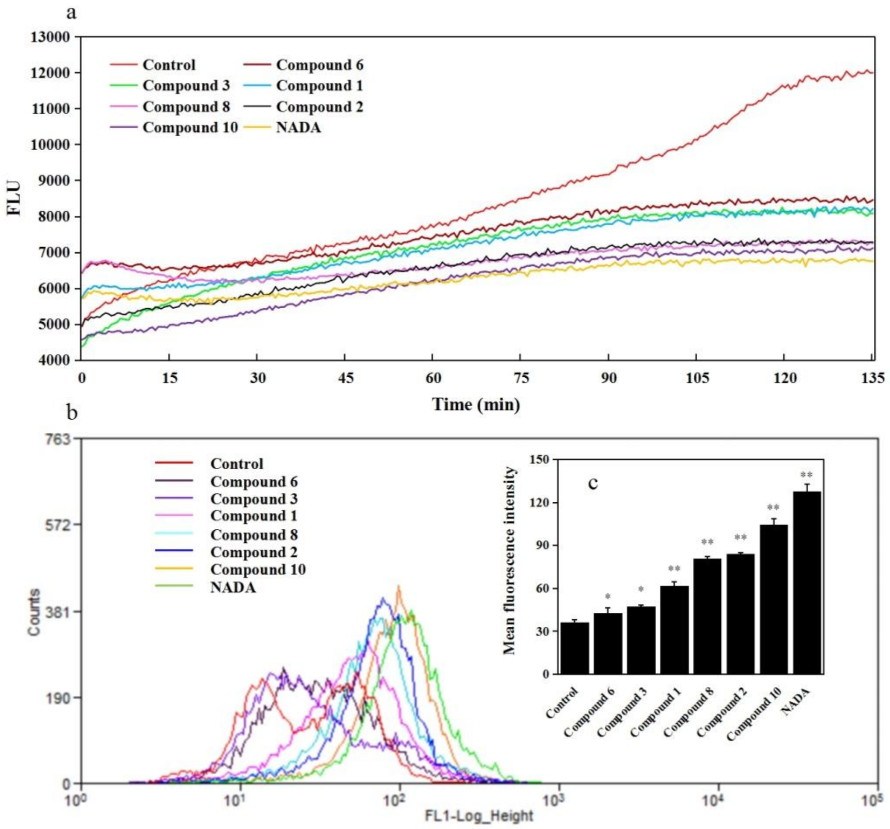

Fig. S5. We compared the extracellular O_2 consumption rate of HeLa cells in the presence of compound **1, 2, 3, 6, 8, 10,** and NADA. These tested compounds could inhibit the mitochondrial respiration of HeLa cells (a), and the inhibition activity had a positive correlation with their cytotoxicity. These compounds could also increase ROS generation (b and c), and the generation of ROS also had a positive correlation with their mitochondrial respiration inhibition and cytotoxicity.

(a) Compound **1, 2, 3, 6, 8, 10** and NADA inhibit the extracellular O_2 consumption of HeLa cells. HeLa cells were seeded 40,000/well. After 24 h, the compounds (4 μM) were added and the inhibition on the mitochondrial respiration was assayed using the extracellular O_2 consumption assay kit (Abcam, Cambridge, USA), according to the manufacture's protocols. (b) Compound **1, 2, 3, 6, 8, 10** and NADA increase ROS generation in HeLa cells. HeLa cells were treated these compounds (0.2 μM) for 5 h, and the intracellular ROS levels were detected by flow cytometry using CM-H2DCFDA probes (Beyotime Biotechnology, Guangzhou, China). (c) Histogram shows the increase of the DCF fluorescence after treatment with these compounds. Values represent the means ± SD. * $P < 0.05$, ** $P < 0.01$ versus control.

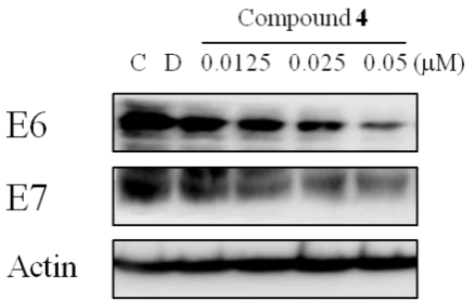

Fig. S6. Compound **4** induces E6/E7 viral oncoproteins degradation in aconcentration dependent manner. HeLa cells were treated with the indicated concentrations of compound **4** for 24 h, and the expression levels of E6/E7 were assessed by western blotting. C, control group; D, DMSO vehicle control.

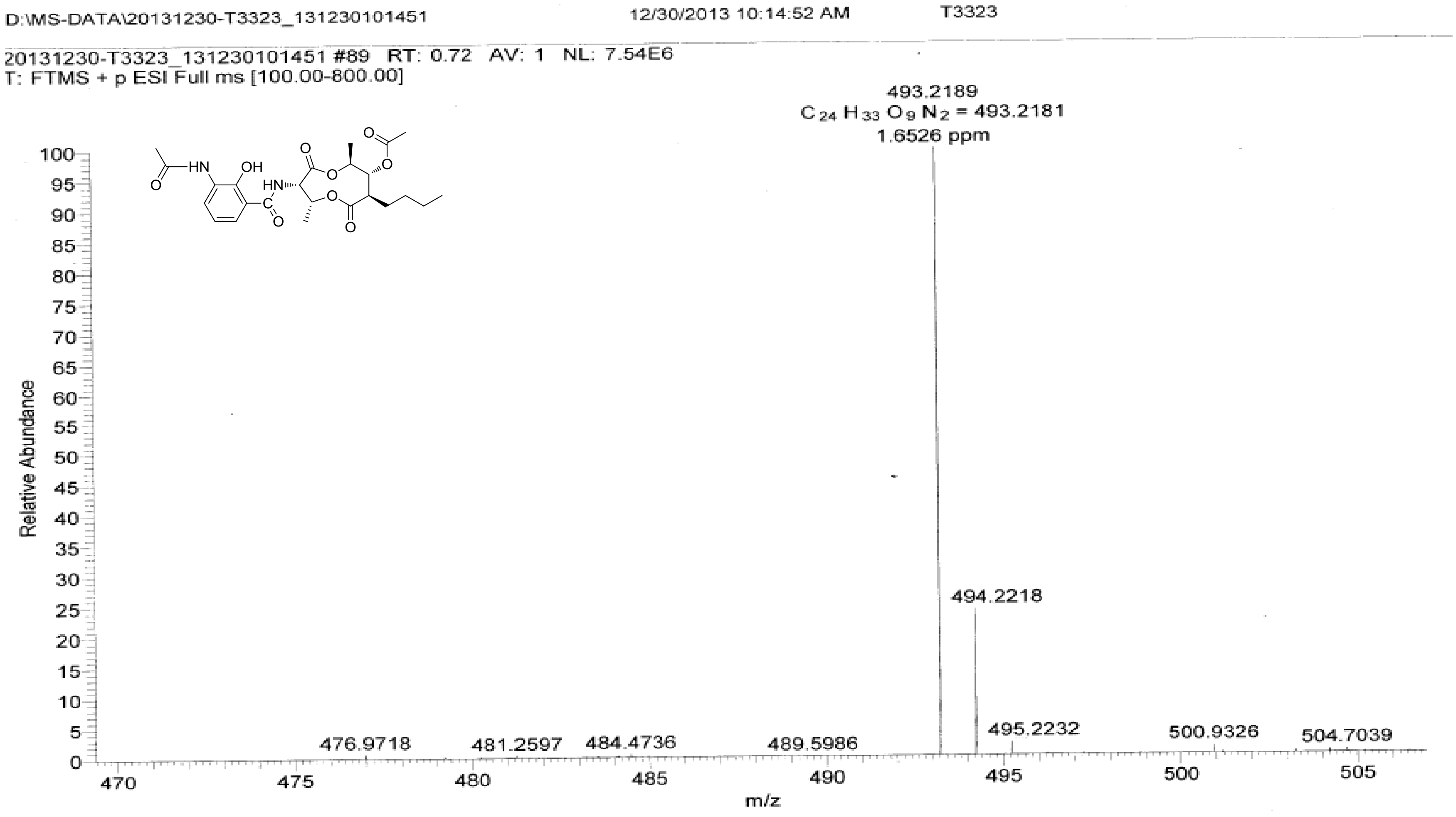

Fig. **S7**. HR-ESI-MS of antimycin E (**1**)

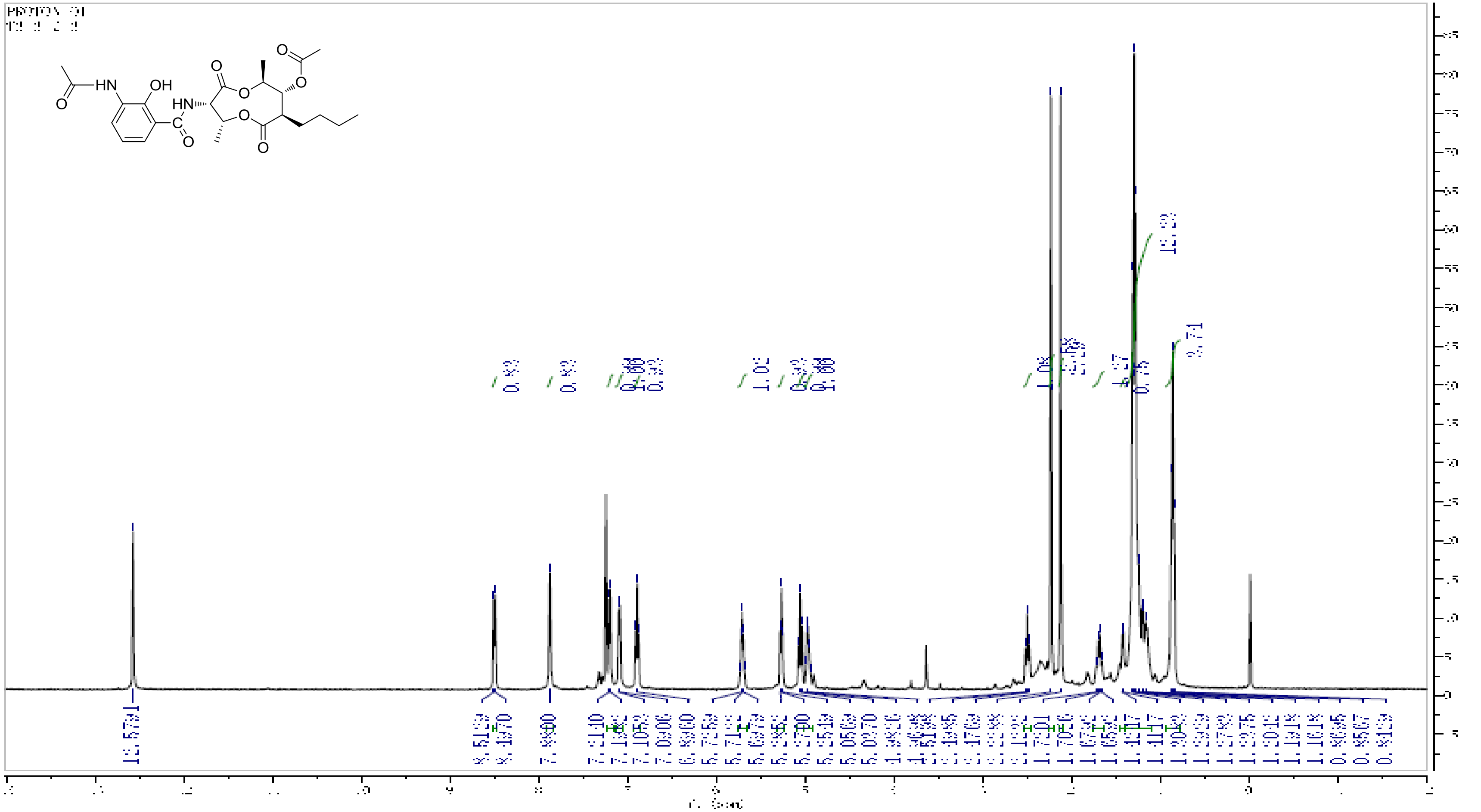

Fig. **S8**. ^{1}H-NMR spectrum (500 MHz) of antimycin E (**1**) in $CDCl_3$

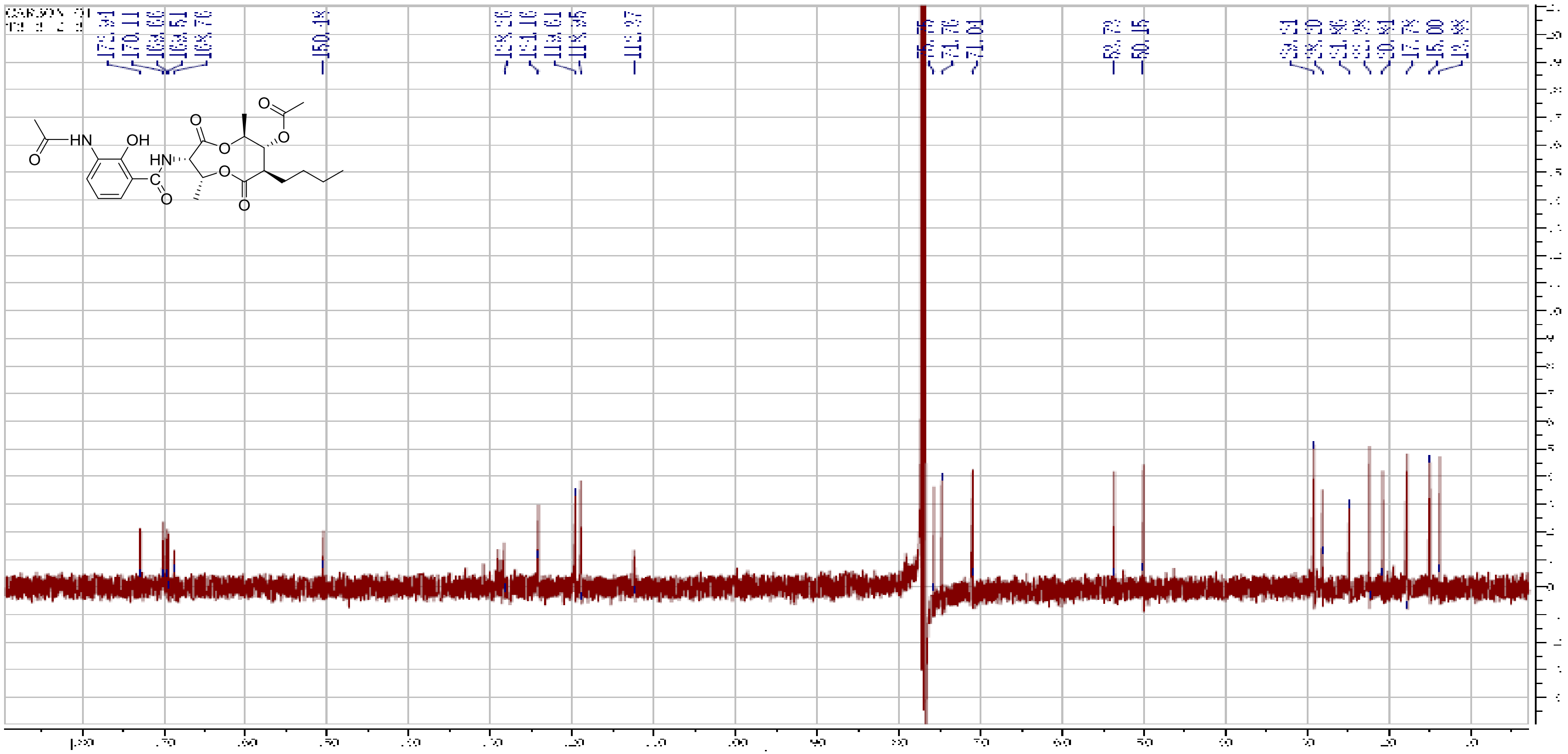

Fig. S9. ^{13}C-NMR spectrum (125 MHz) of antimycin E (**1**) in $CDCl_3$

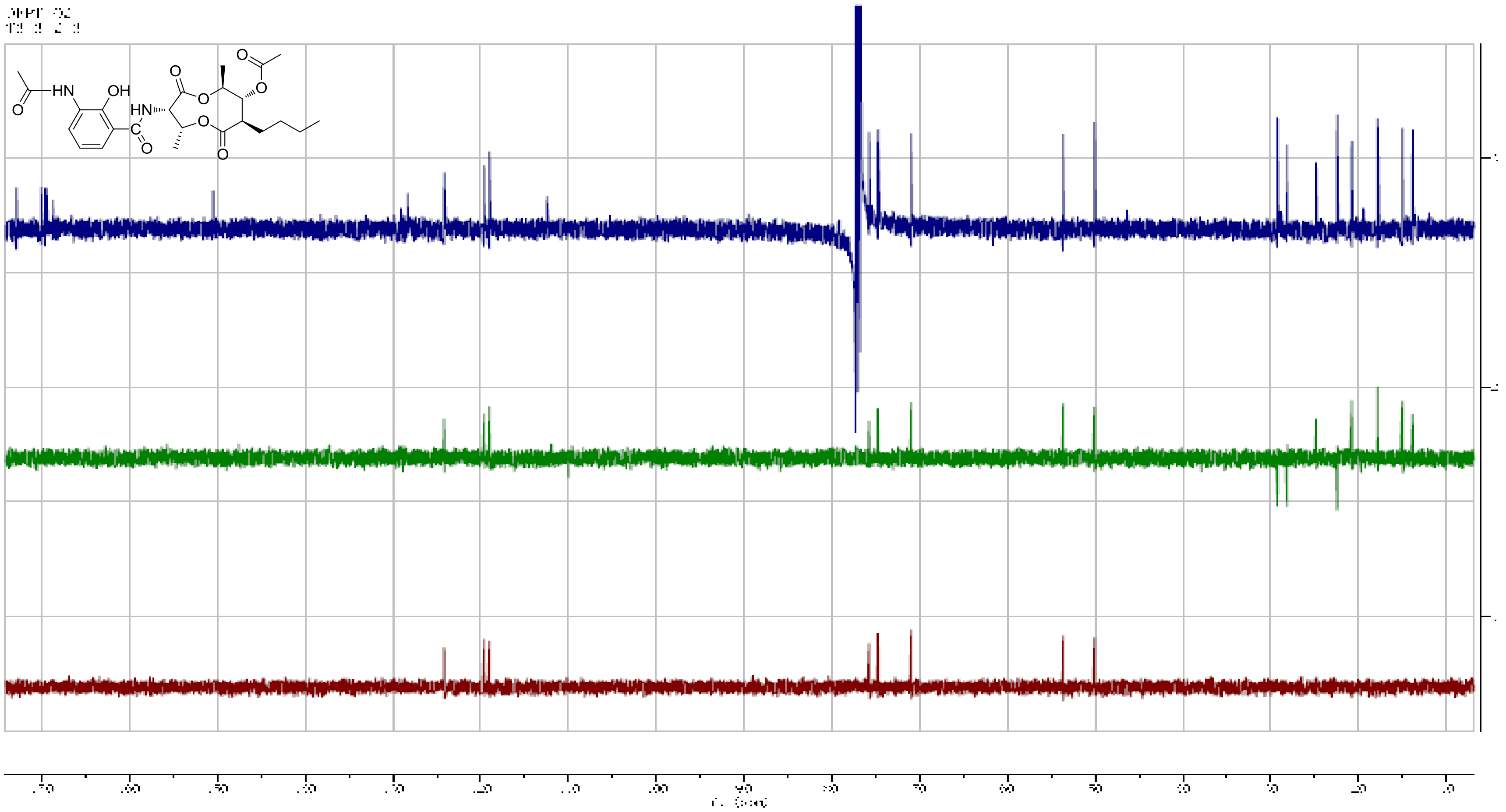

Fig. S10. DEPT spectrum (125 MHz) of antimycin E (**1**) in $CDCl_3$

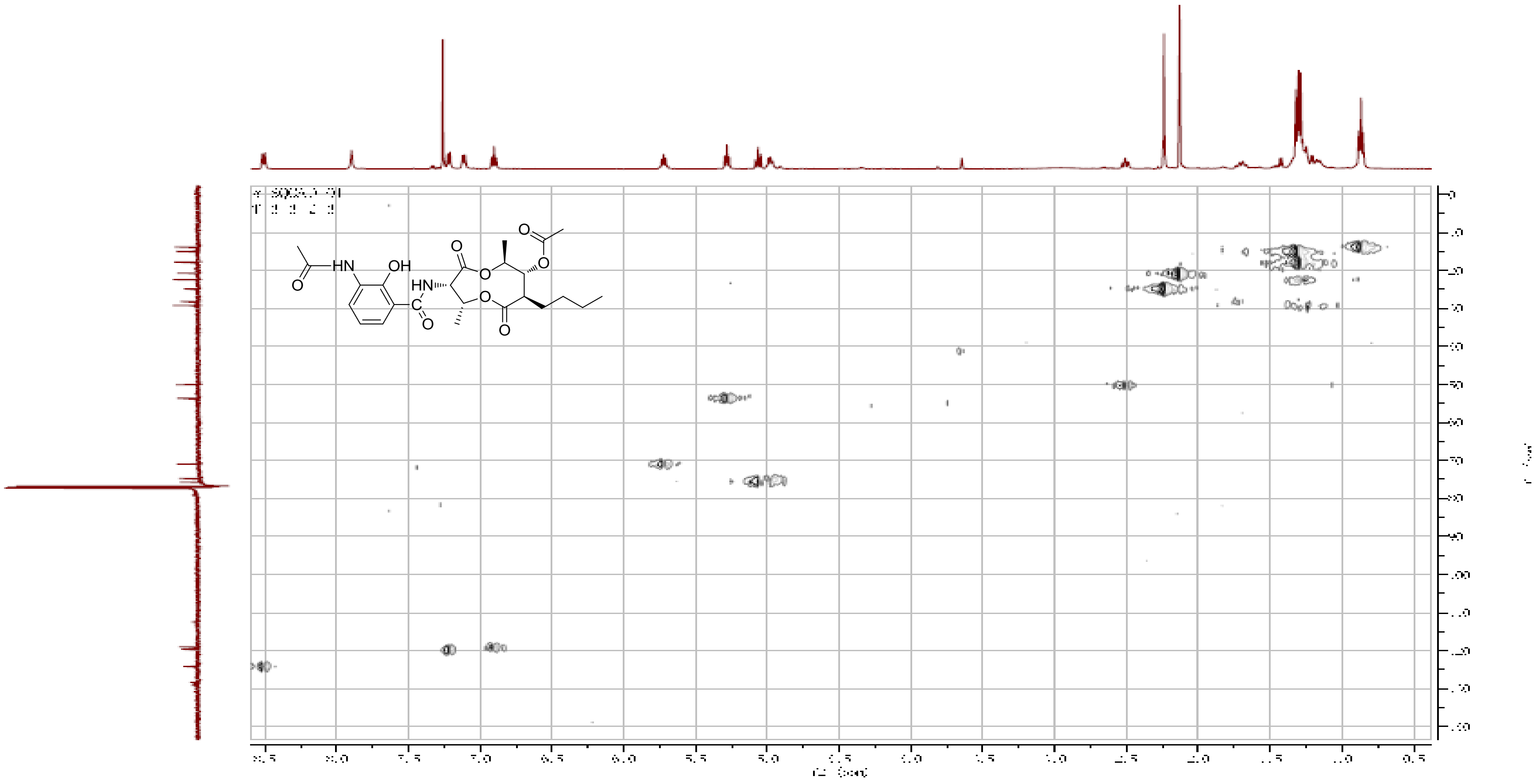

Fig. S11. HSQC spectrum (500 MHz) of antimycin E (**1**) in $CDCl_3$

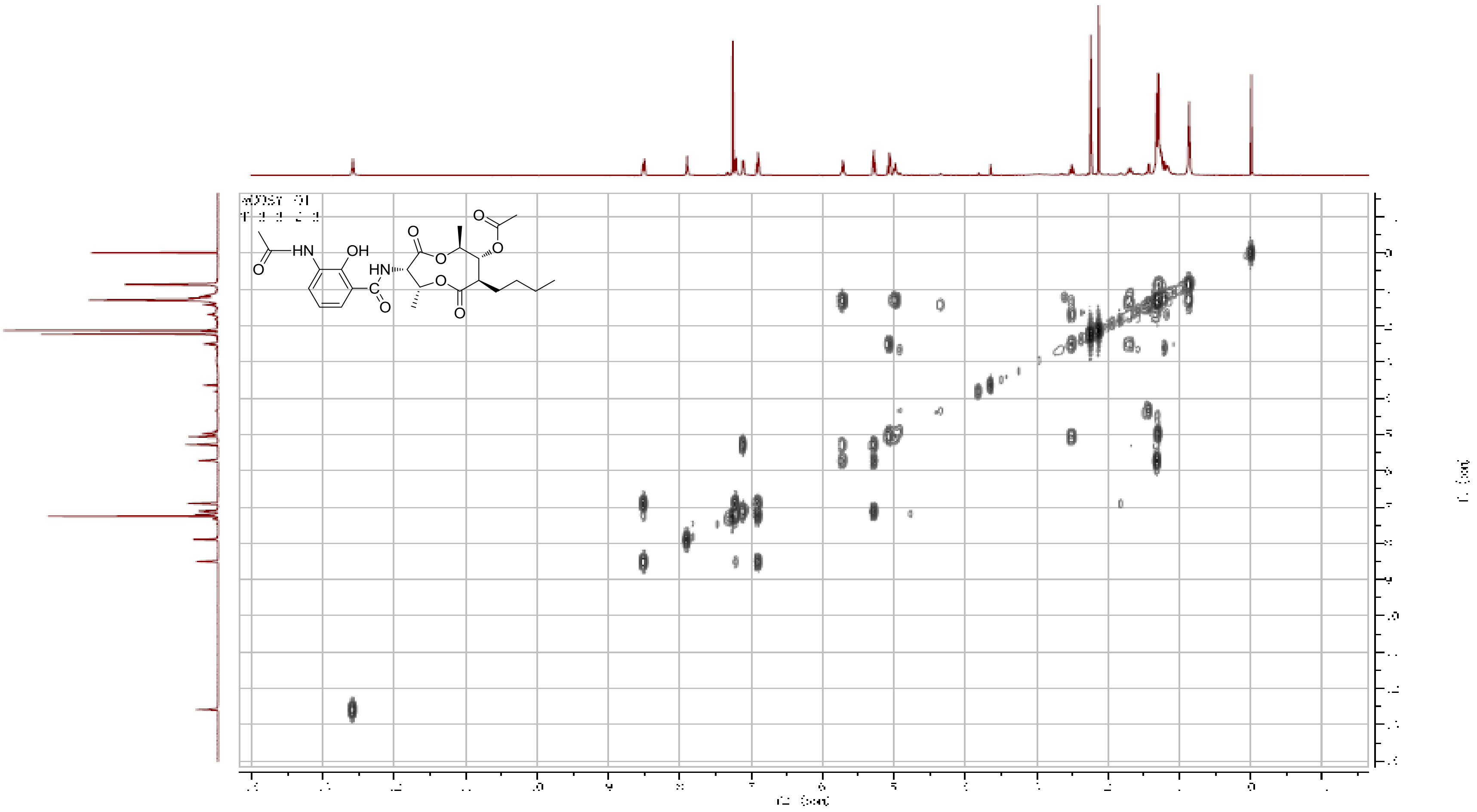

Fig. S12. ^{1}H-^{1}H COSY spectrum (500 MHz) of antimycin E (**1**) in $CDCl_3$

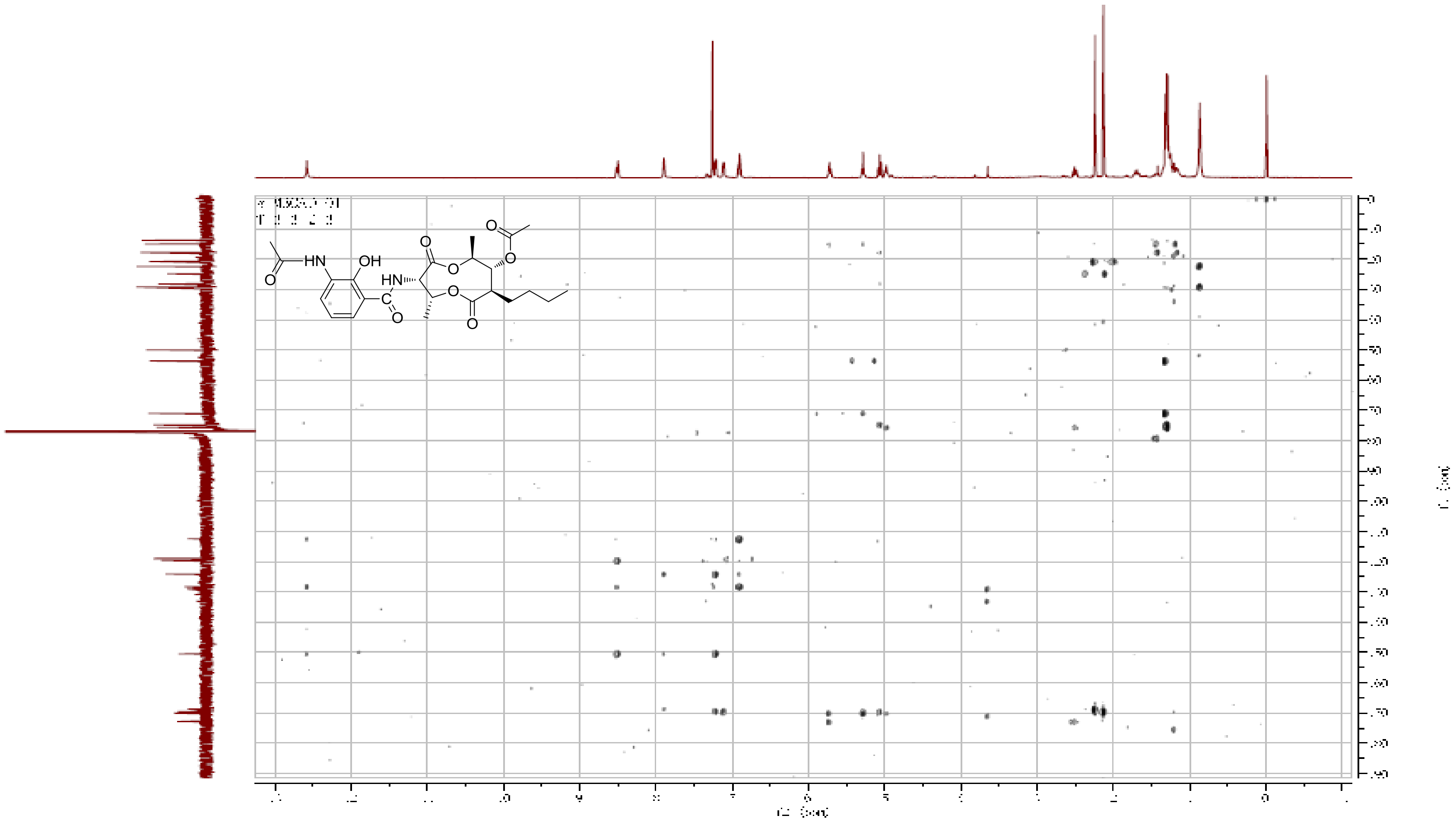

Fig. S13. HMBC spectrum (500 MHz) of antimycin E (**1**) in $CDCl_3$

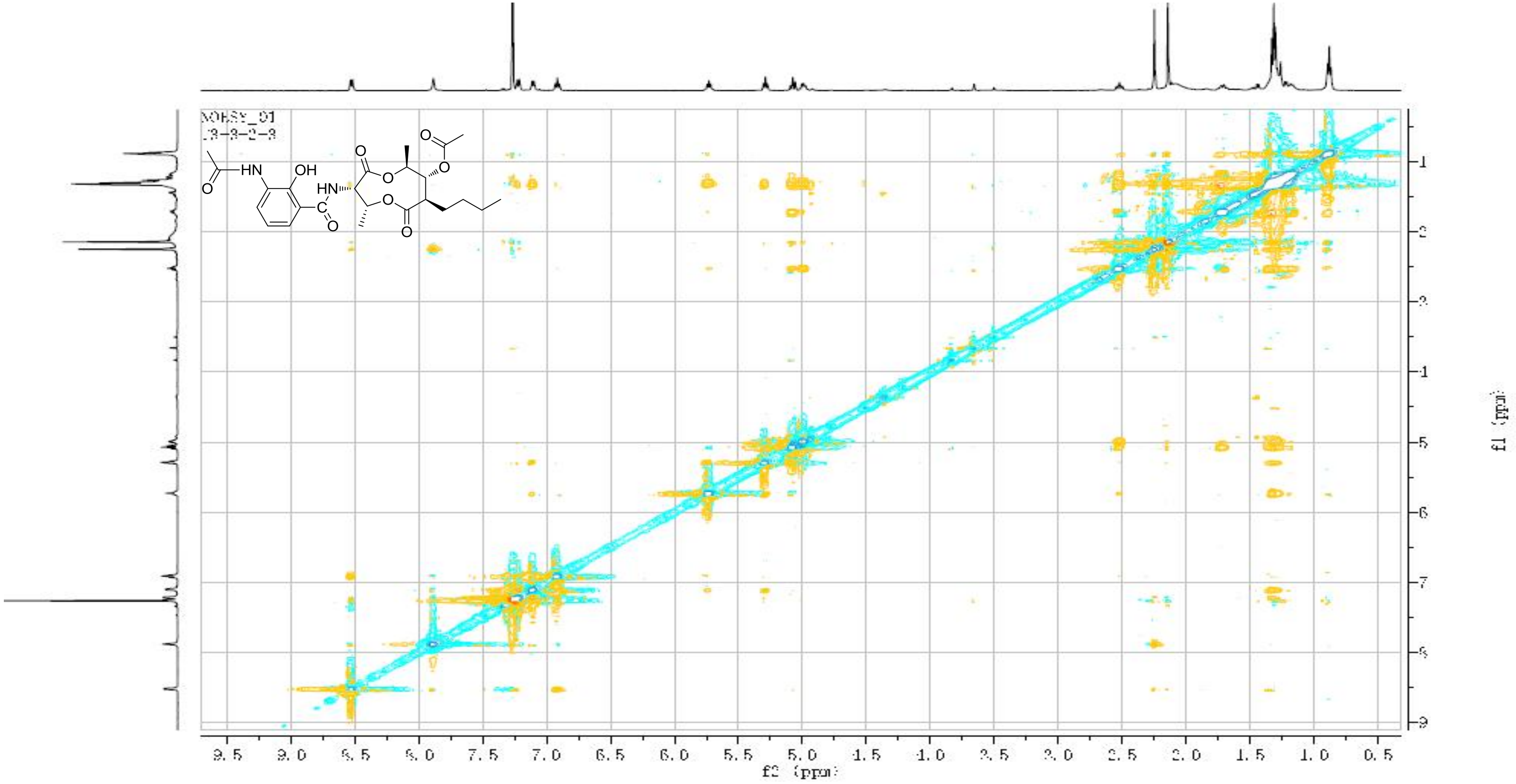

Fig. S14. NOESY spectrum (500 MHz) of antimycin E (**1**) in $CDCl_3$

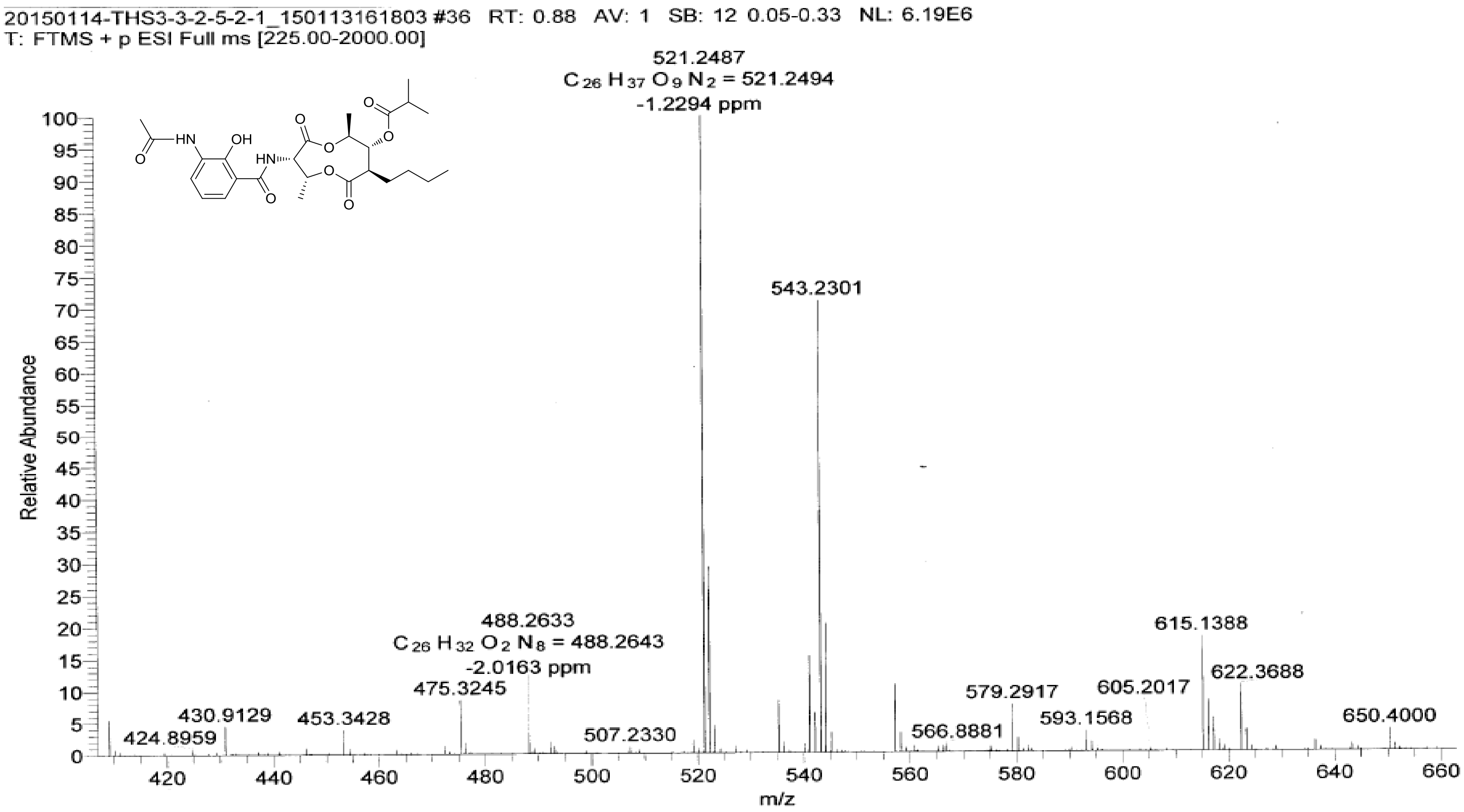

Fig. S15. HR-ESI-MS of antimycin F (**2**)

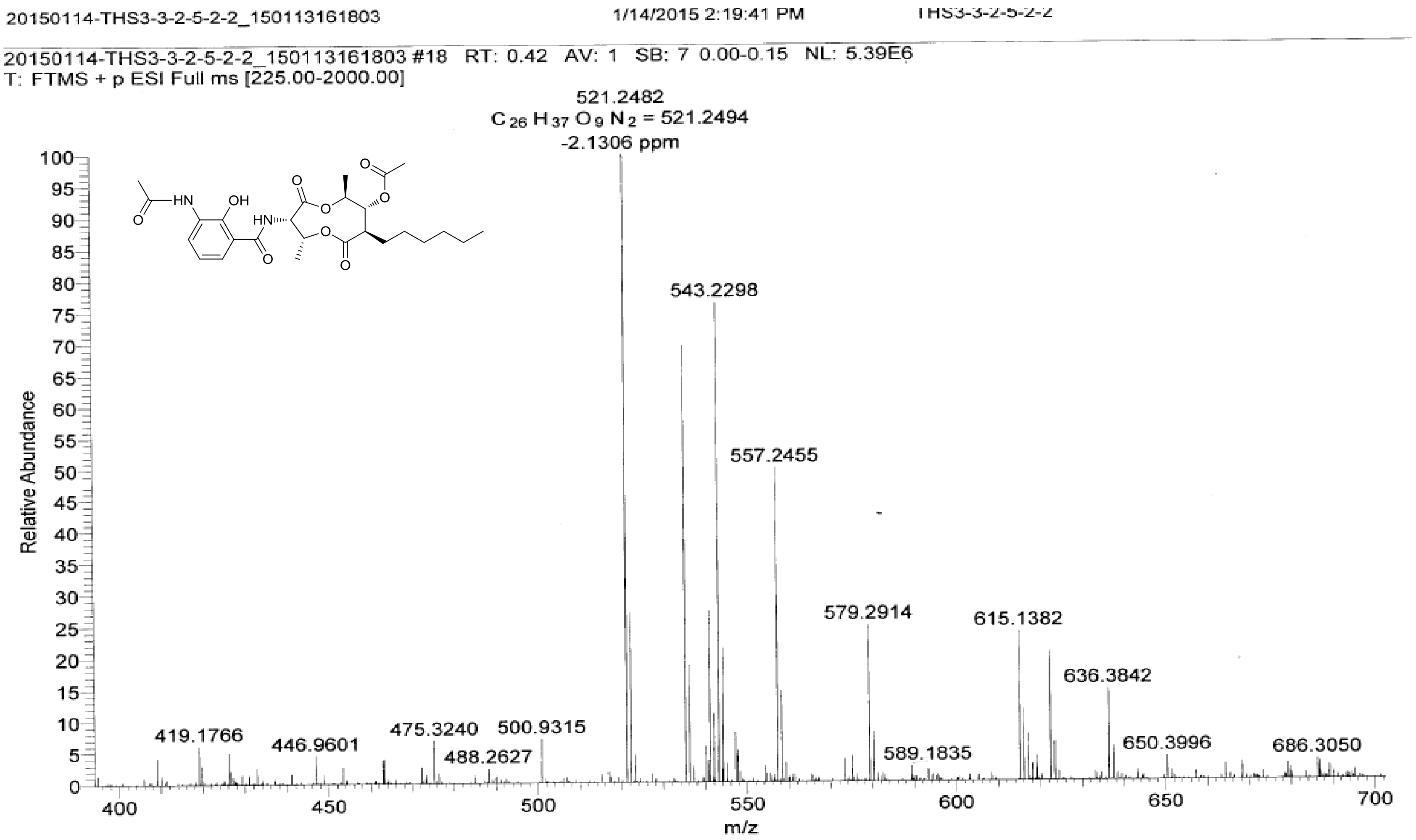

Fig. S16. HR-ESI-MS of antimycin G (**3**)

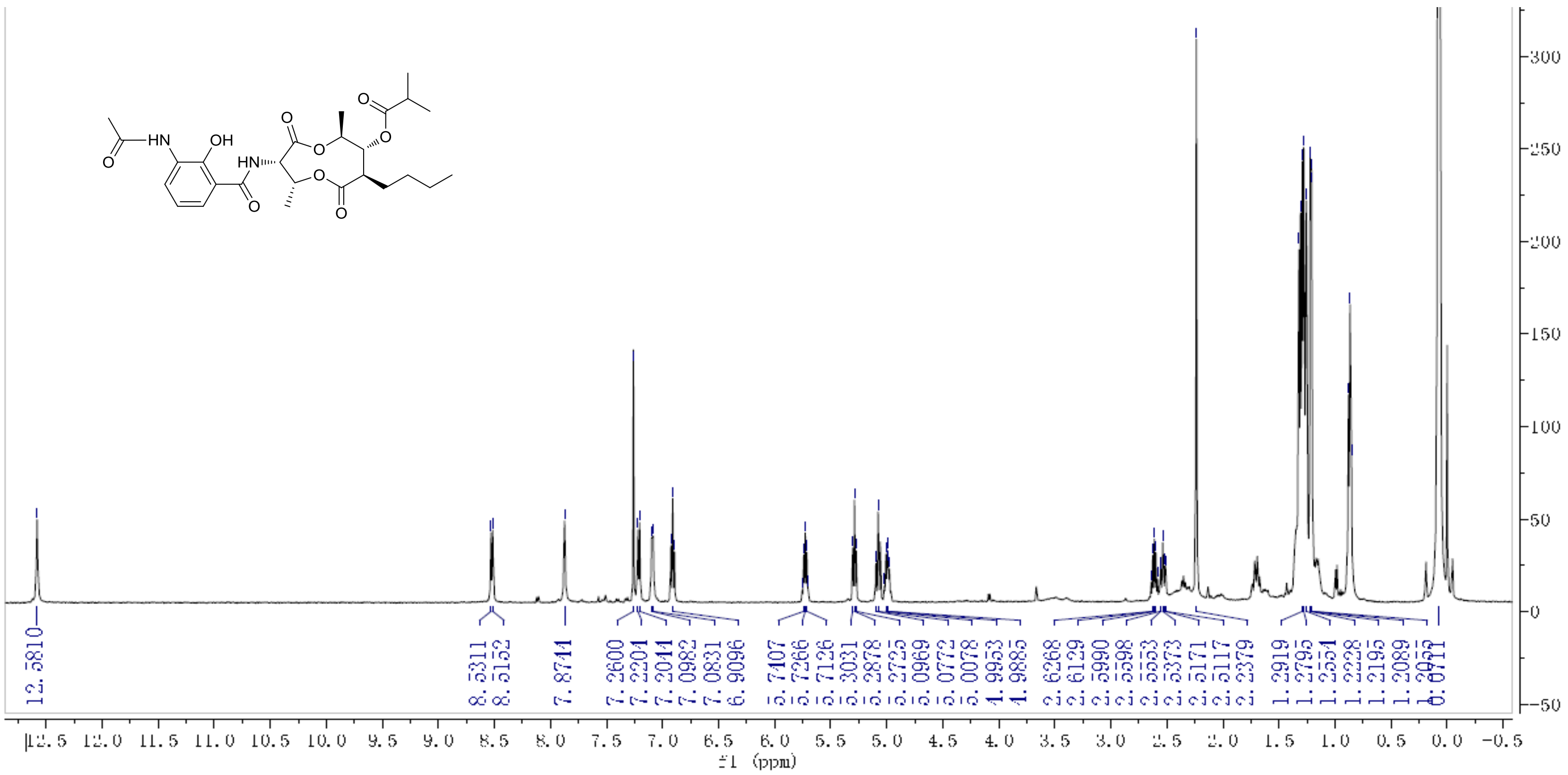

Fig. S17. ^{1}H-NMR spectrum (500 MHz) of antimycin F (**2**) in $CDCl_3$

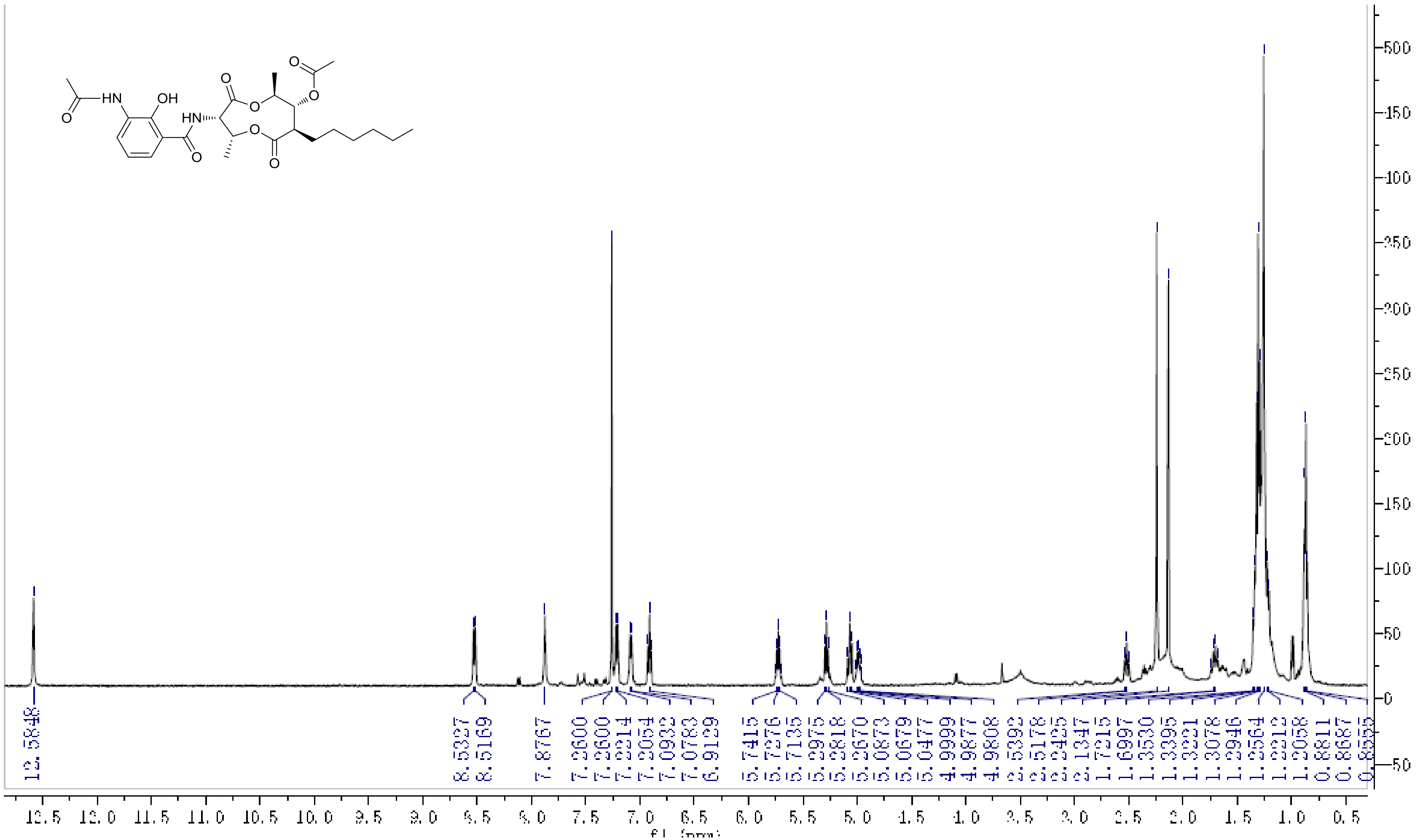

Fig. S18. ^{1}H-NMR spectrum (500 MHz) of antimycin G (**3**) in $CDCl_3$

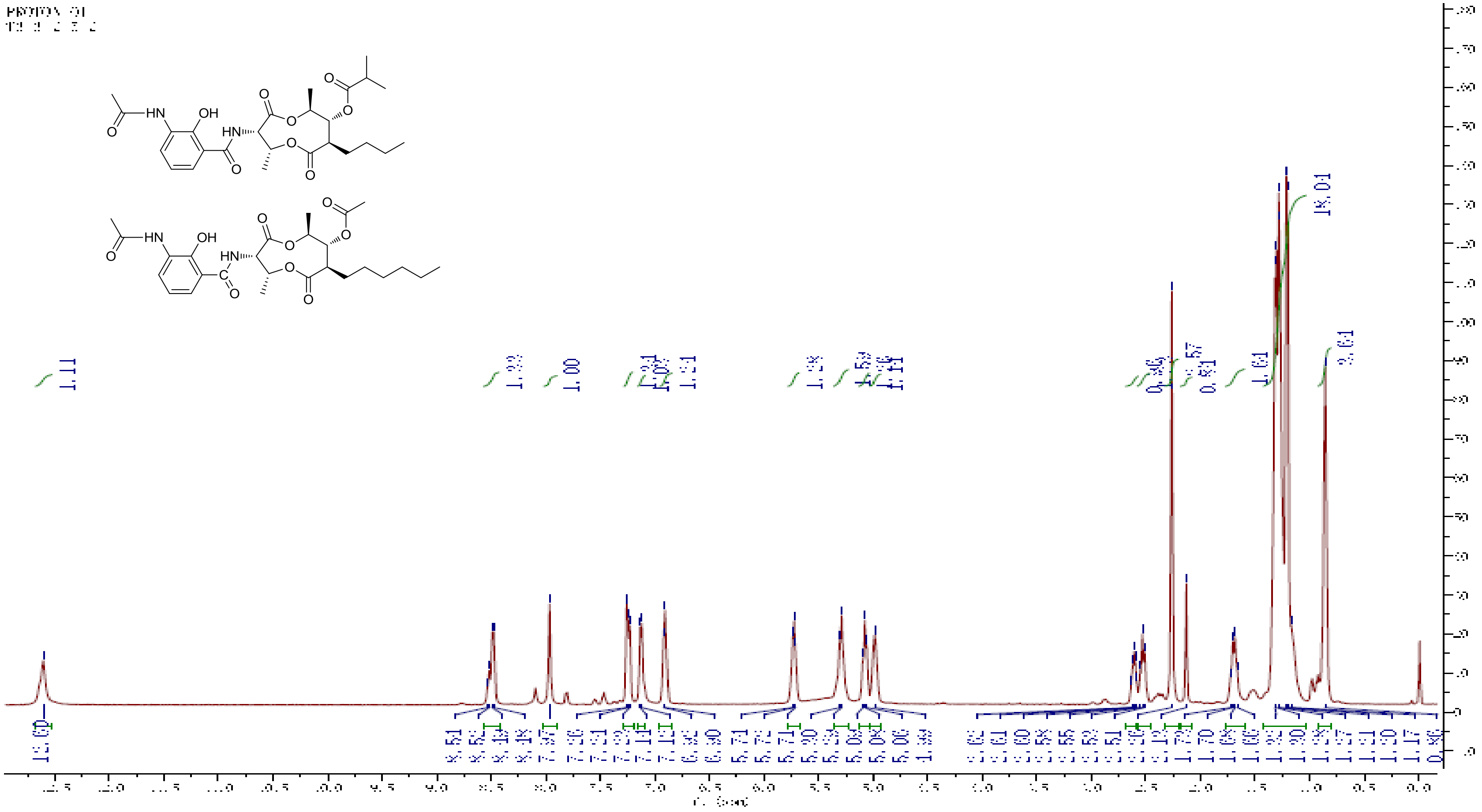

Fig. S19. ^{1}H-NMR spectrum (500 MHz) of antimycins F (**2**) and G (**3**) in $CDCl_3$

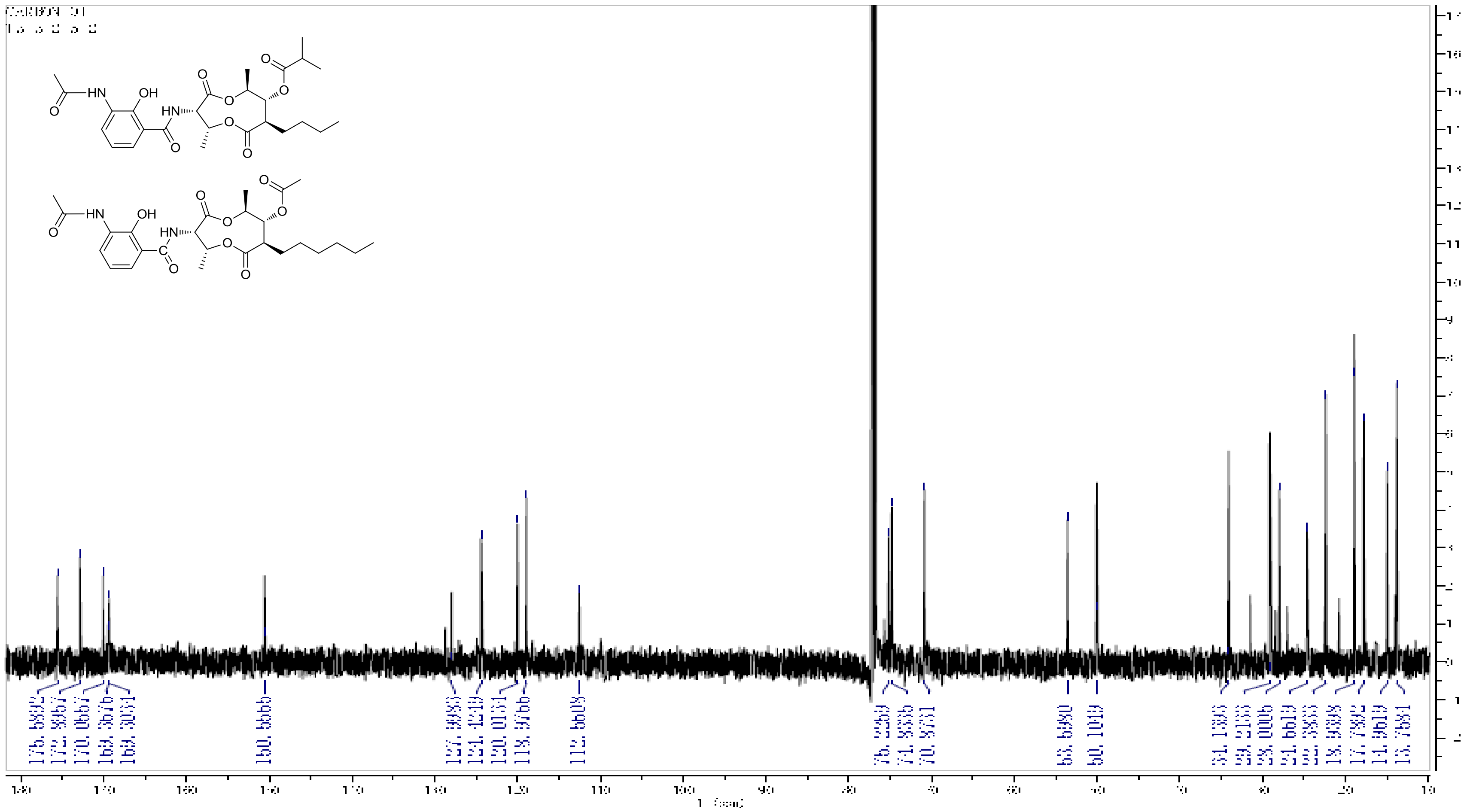

Fig. S20. ^{13}C-NMR spectrum (125 MHz) of antimycins F (**2**) and G (**3**) in $CDCl_3$

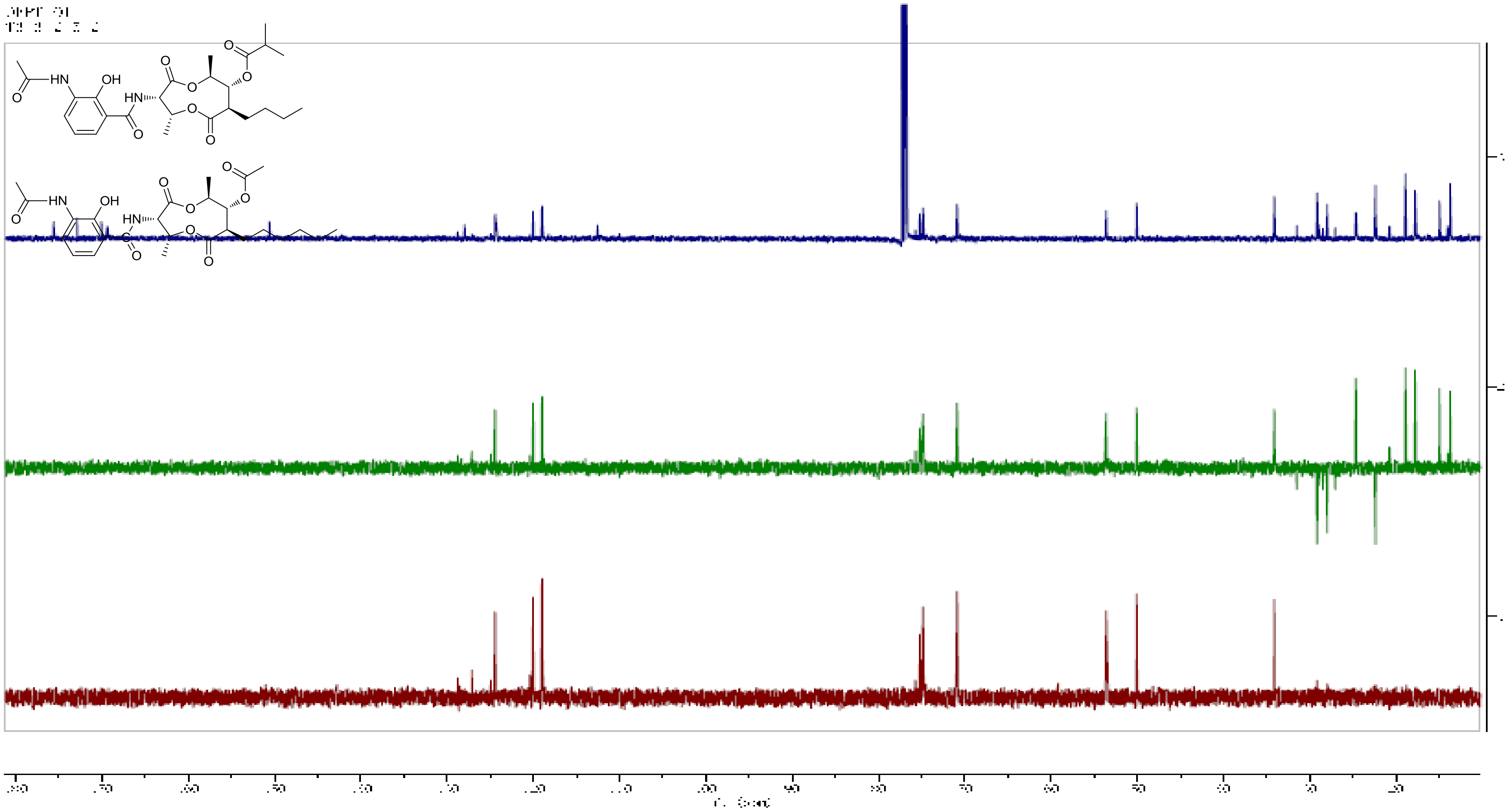

Fig. S21. DEPT spectrum (125 MHz) of antimycins F (**2**) and G (**3**) in $CDCl_3$

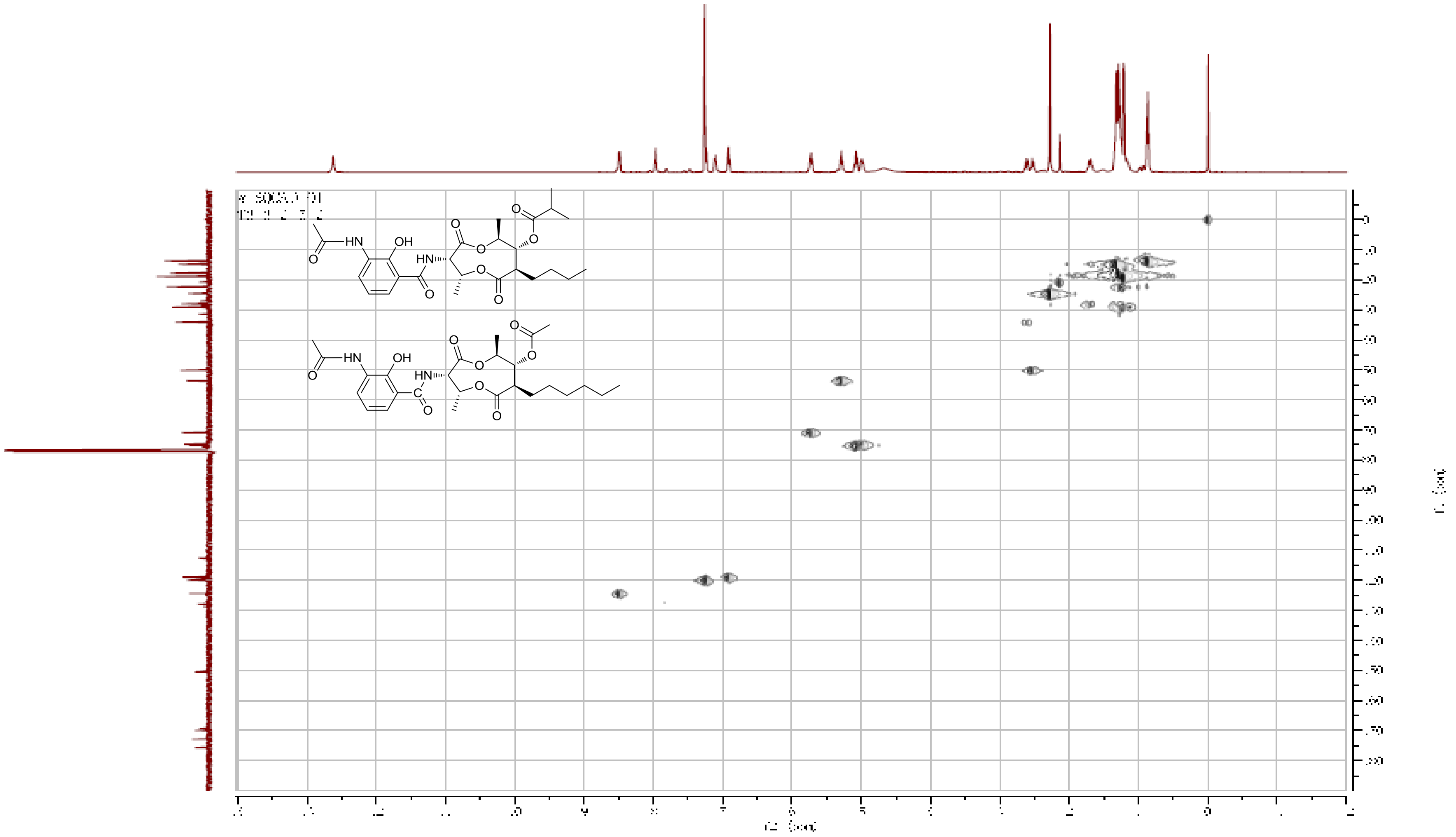

Fig. S22. HSQC spectrum (500 MHz) of antimycins F (**2**) and G (**3**) in $CDCl_3$

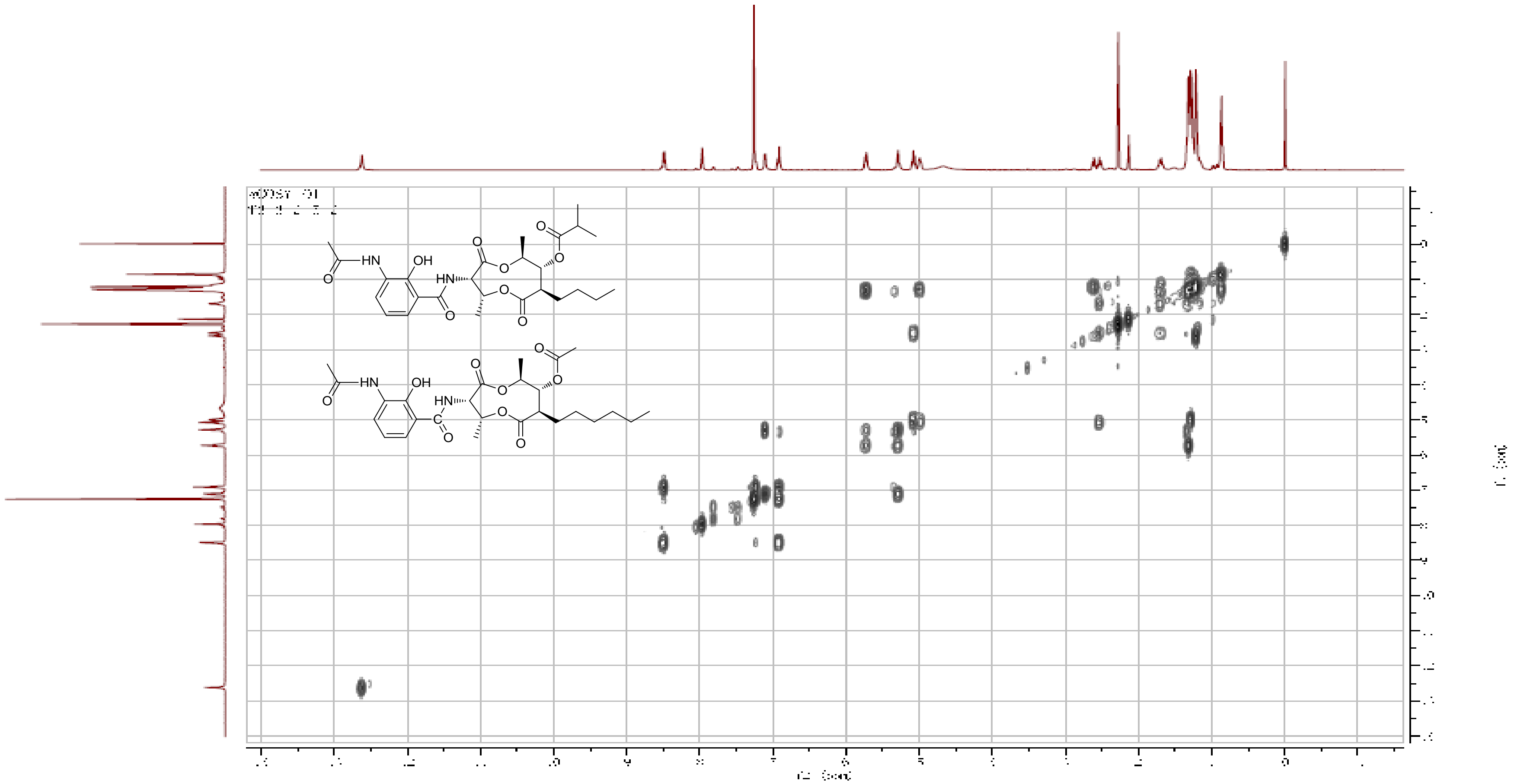

Fig. S23. ^{1}H-^{1}H COSY spectrum (500 MHz) of antimycins F (**2**) and G (**3**) in $CDCl_3$.

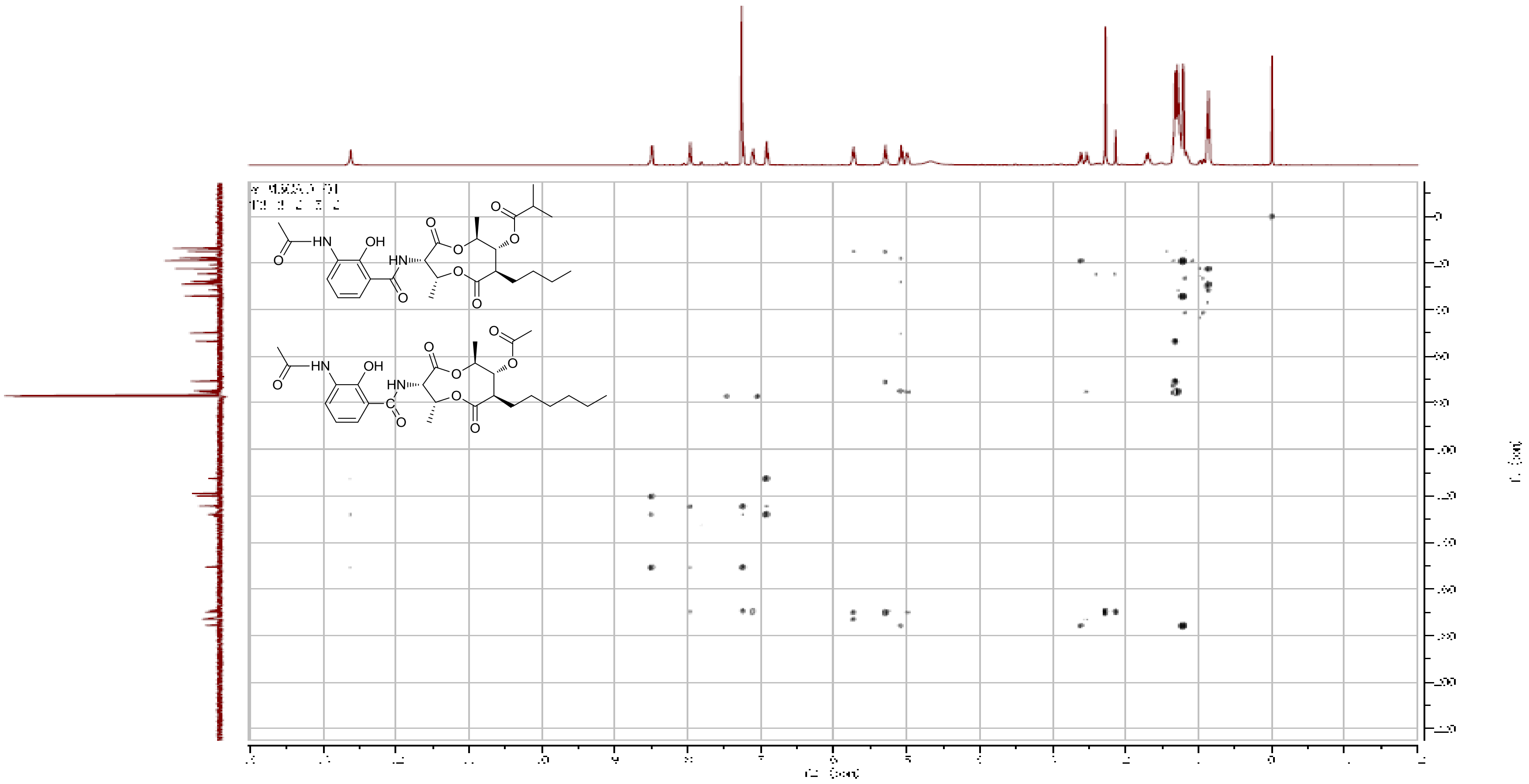

Fig. S24. HMBC spectrum (500 MHz) of antimycins F (**2**) and G (**3**) in $CDCl_3$

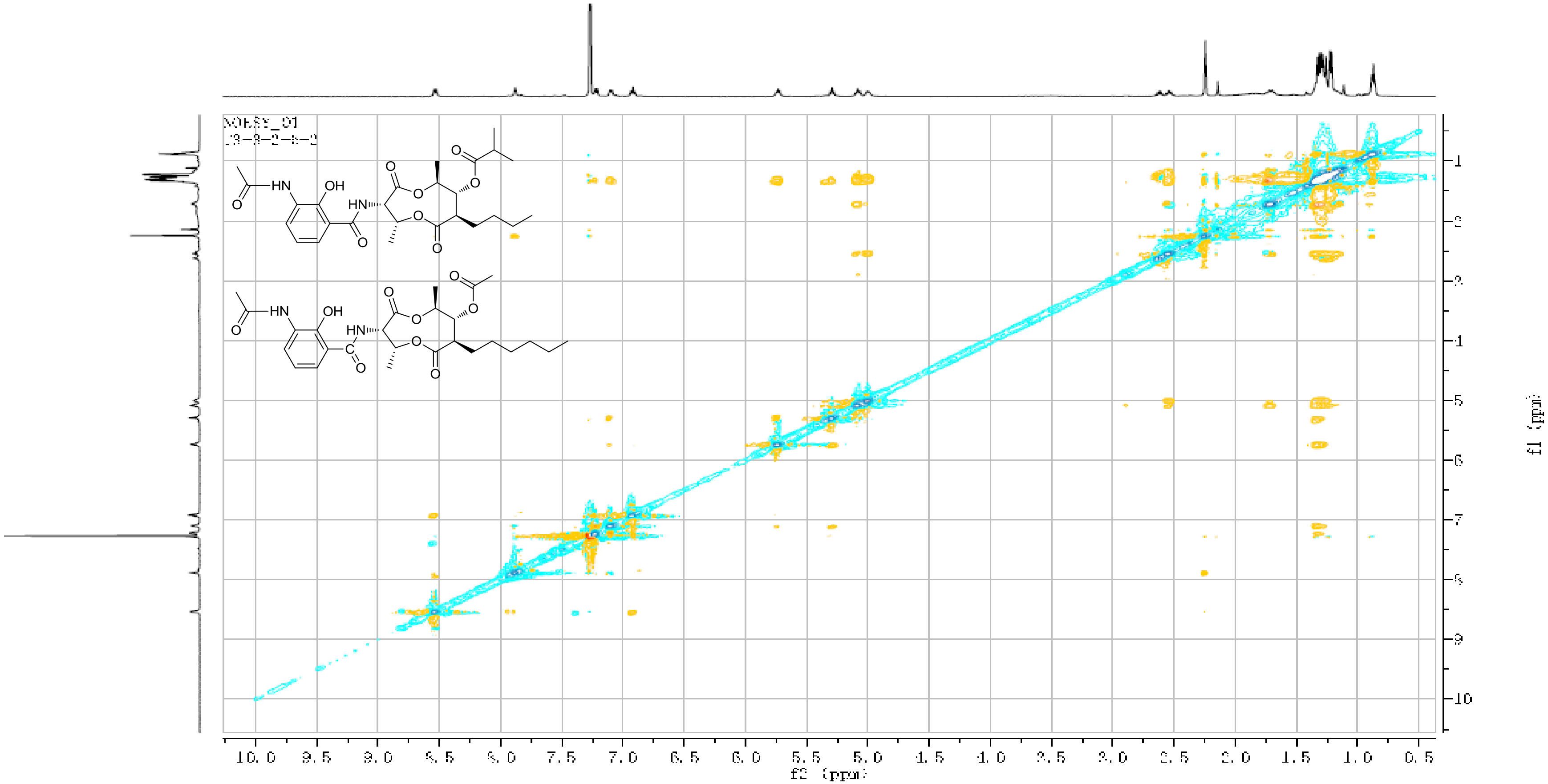

Fig. S25. NOESY spectrum (500 MHz) of antimycins F (**2**) and G (**3**) in $CDCl_3$

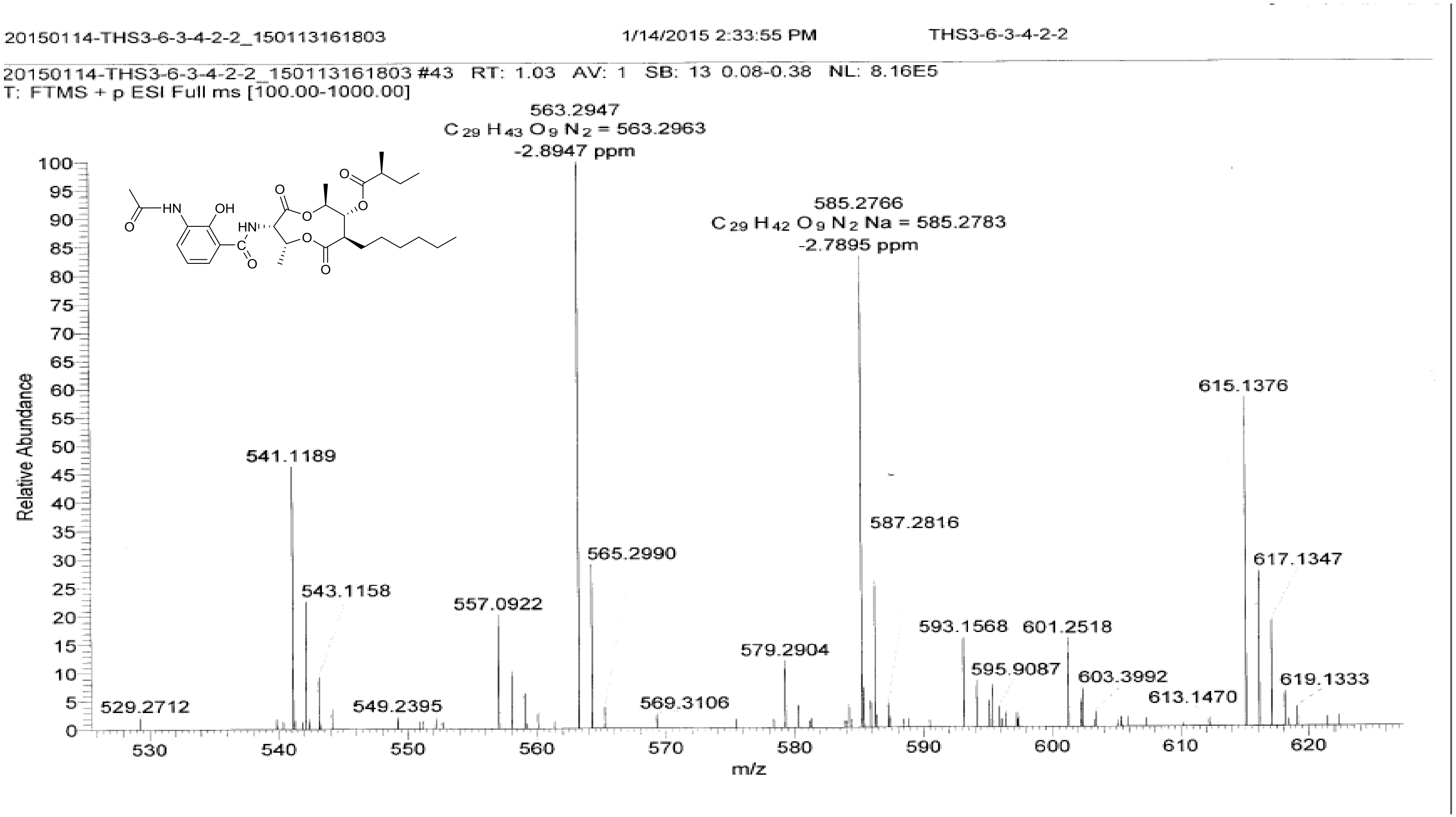

Fig. S26. HR-ESI-MS of antimycin H (**4**)

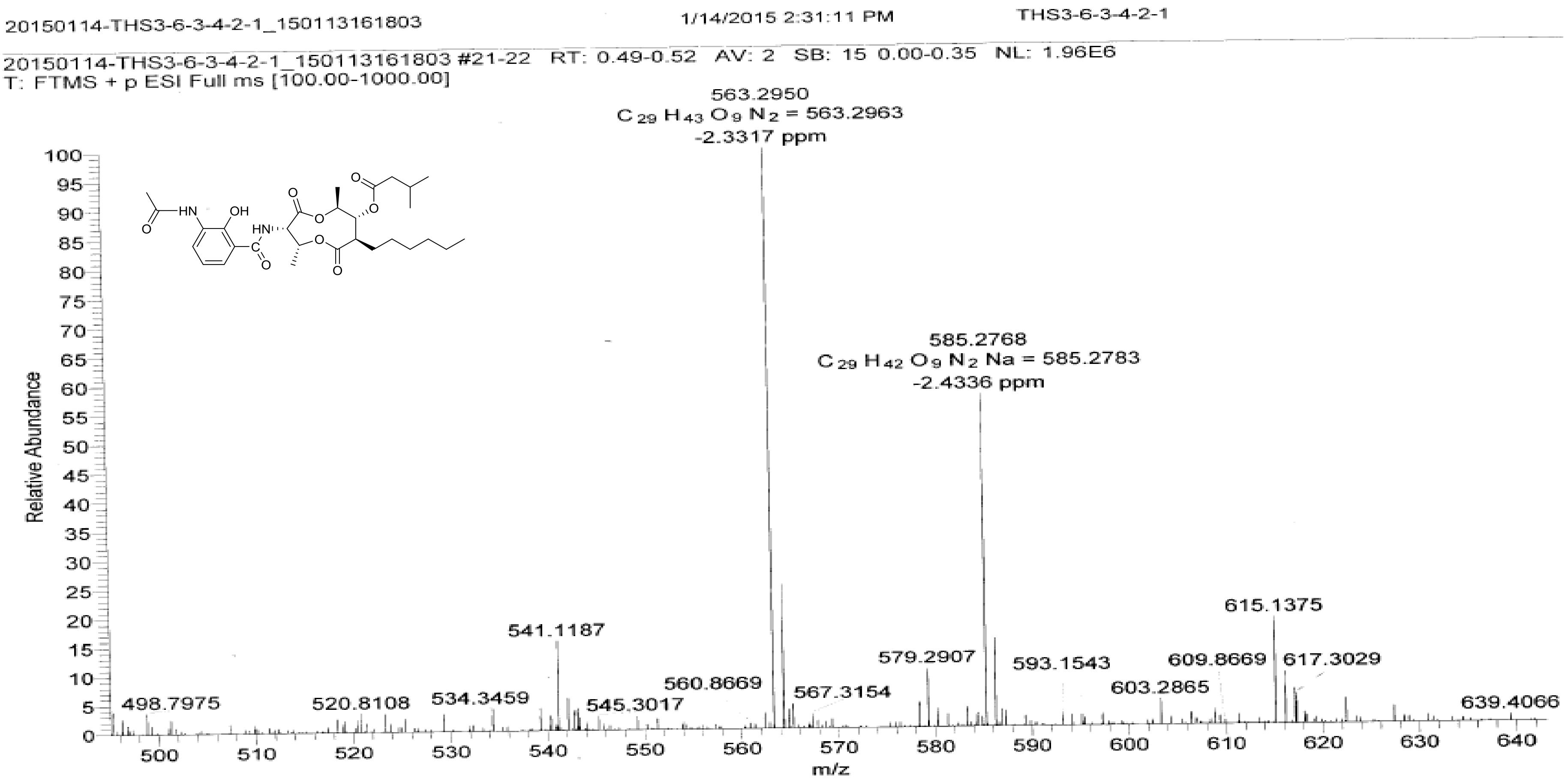

Fig. S27. HR-ESI-MS of NADA (**5**)

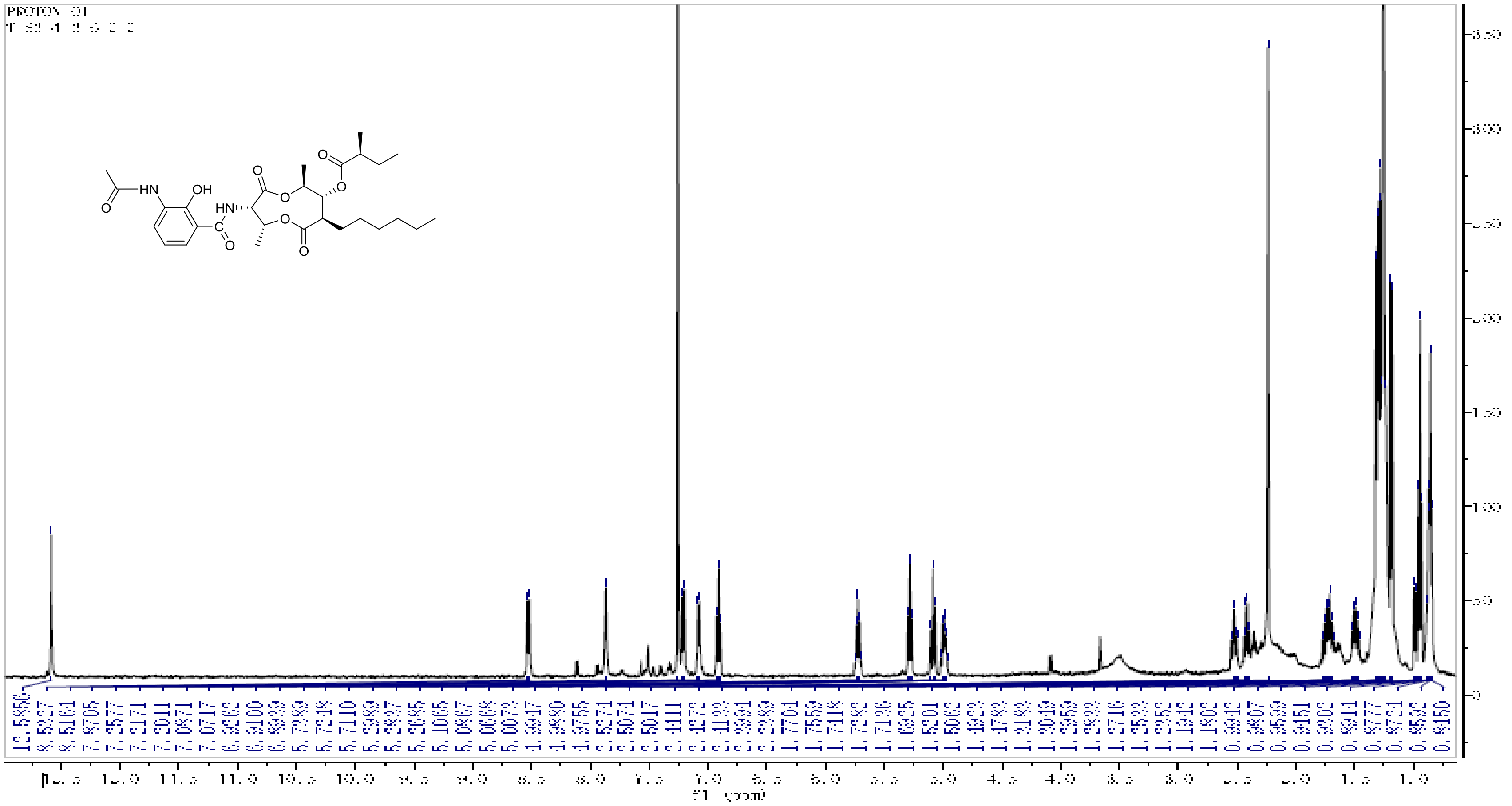

Fig. S28. ^{1}H-NMR spectrum (500 MHz) of antimycin H (**4**) in $CDCl_3$

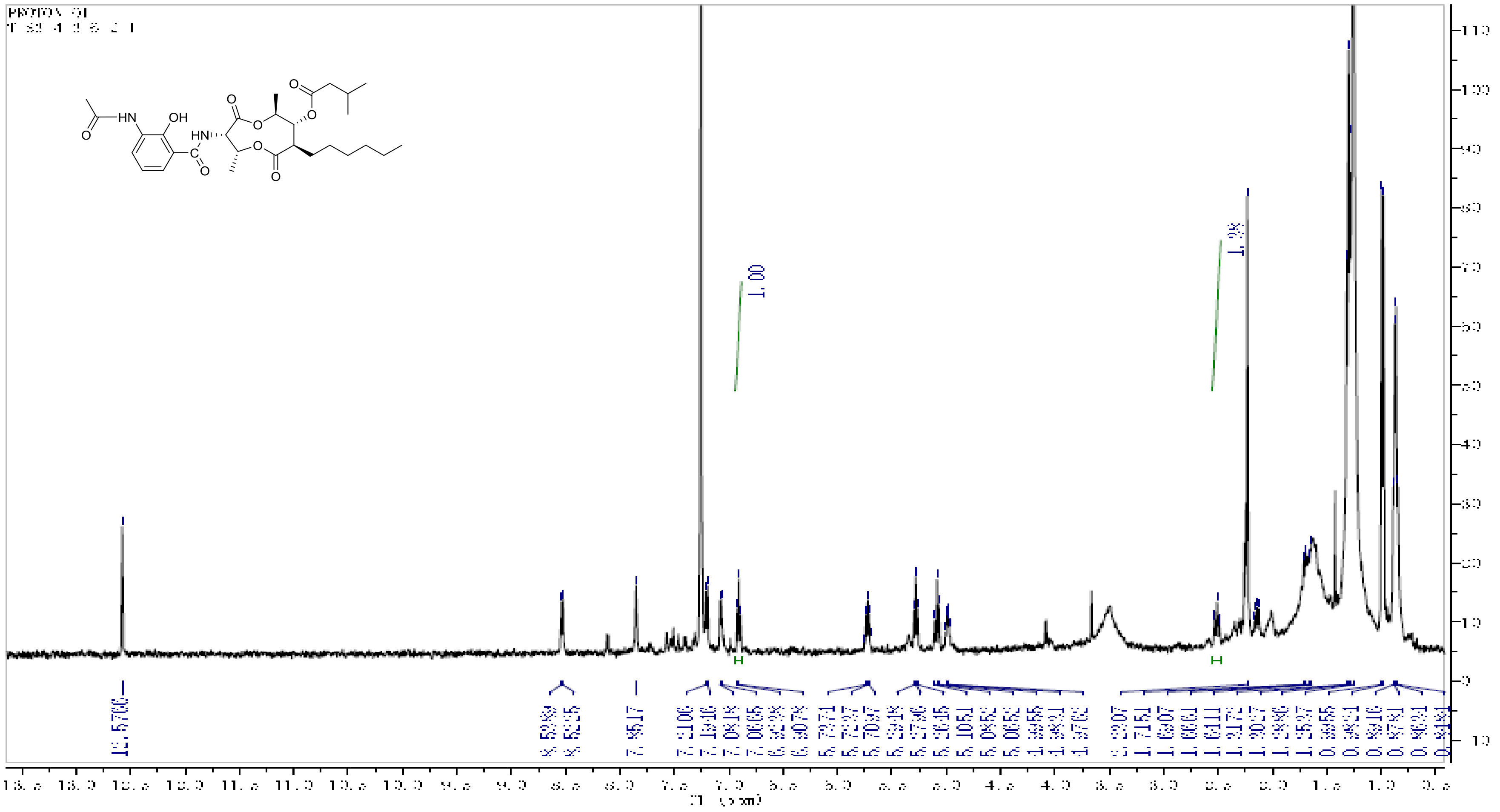

Fig. S29. ^{1}H-NMR spectrum (500 MHz) of NADA (**5**) in $CDCl_3$

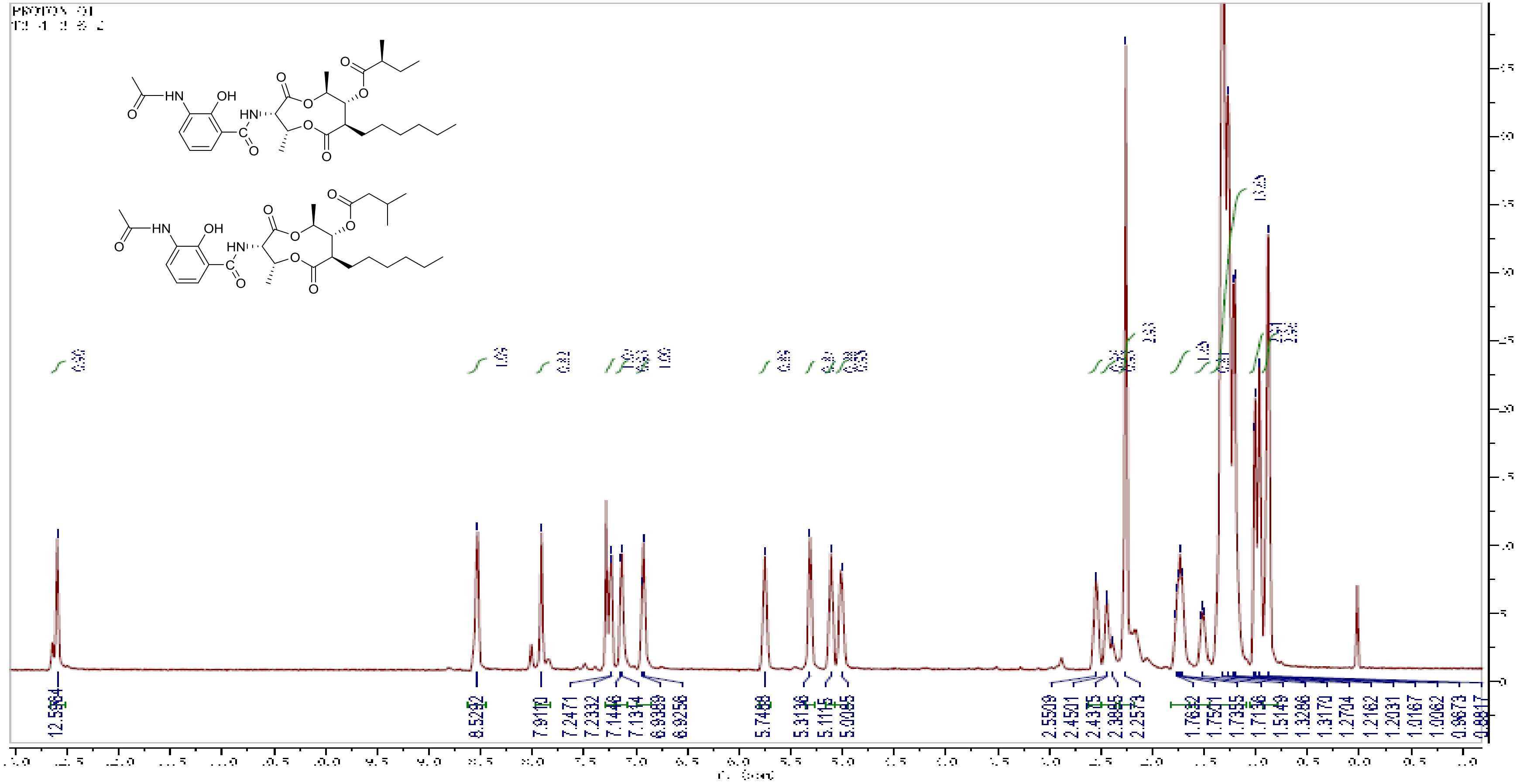

Fig. S30. ^{1}H-NMR spectrum (500 MHz) of antimycin H (**4**) and NADA (**5**) in $CDCl_3$

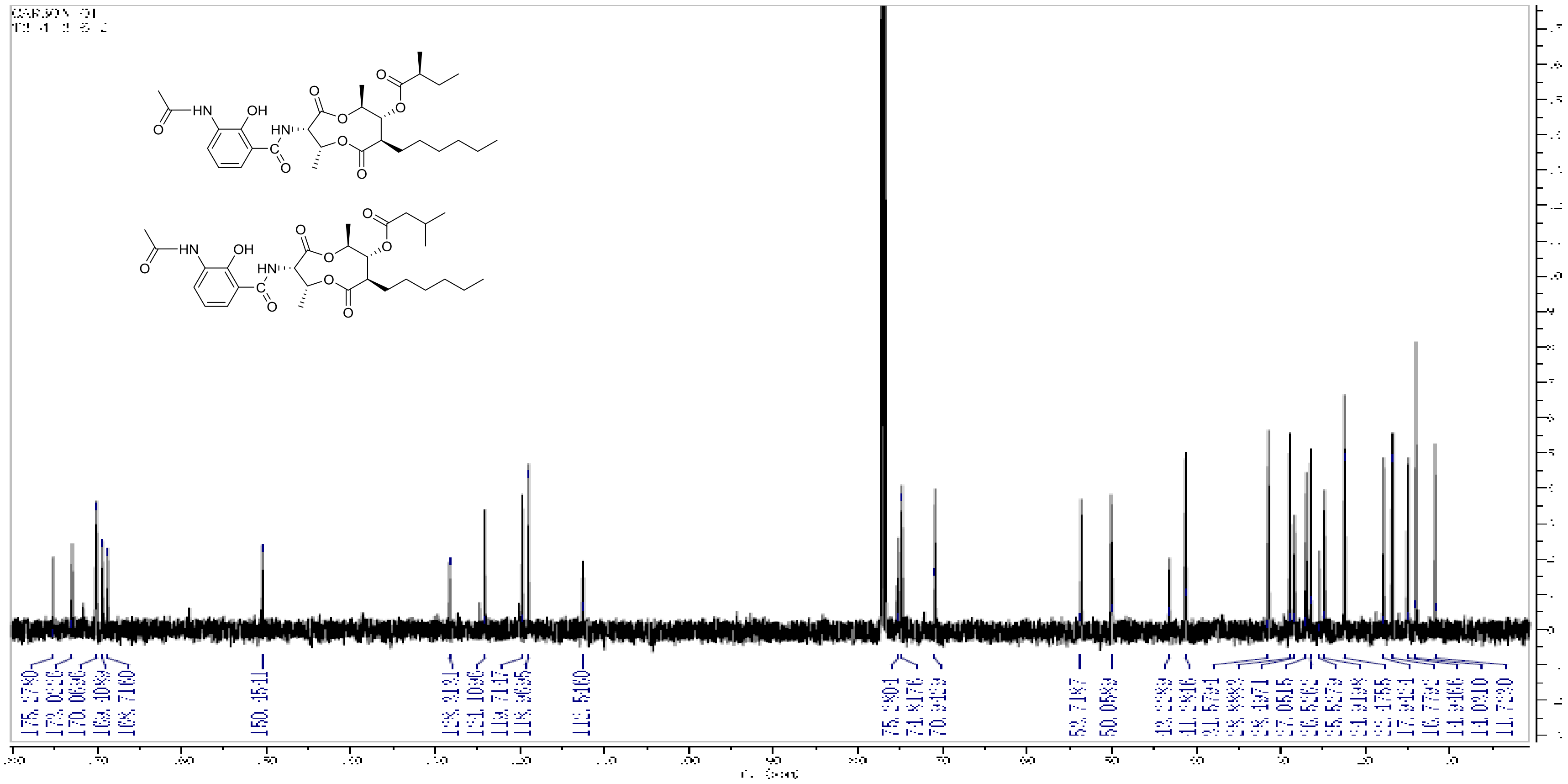

Fig. S31. ^{13}C-NMR spectrum (125 MHz) of antimycin H (**4**) and NADA (**5**) in $CDCl_3$

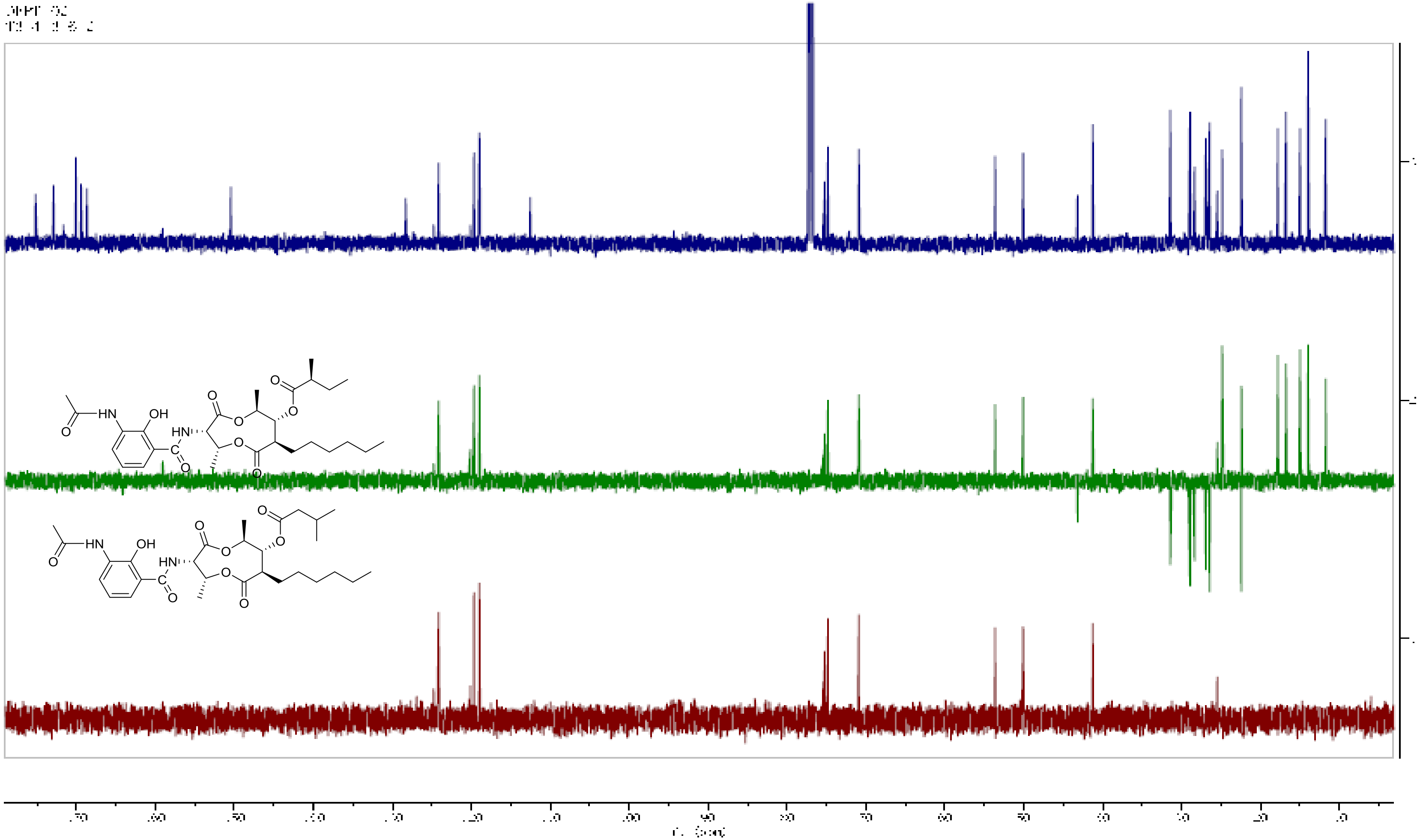

Fig. S32. DEPT spectrum (125 MHz) of antimycin H (**4**) and NADA (**5**) in $CDCl_3$

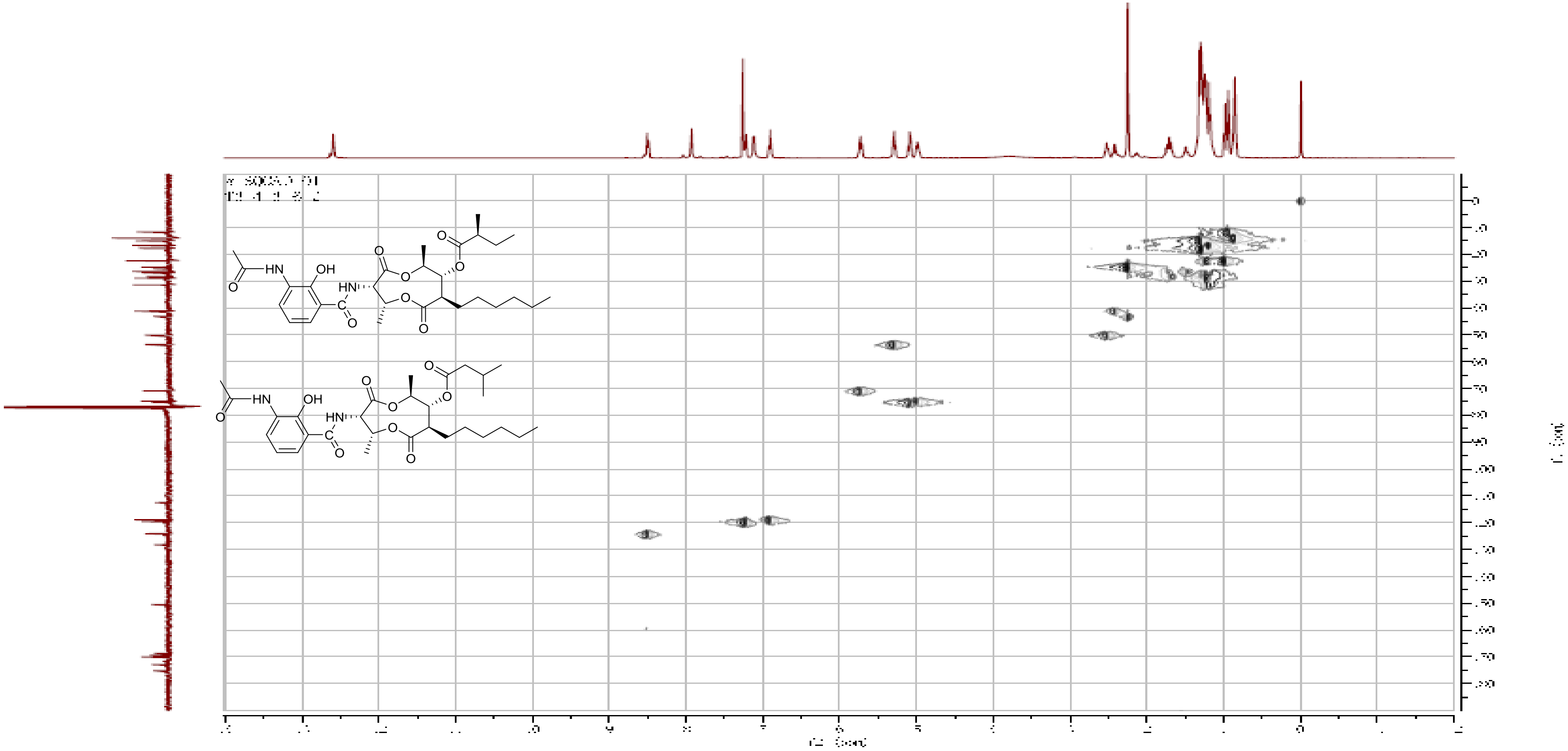

Fig. S33. HSQC spectrum (500 MHz) of antimycin H (**4**) and NADA (**5**) in $CDCl_3$

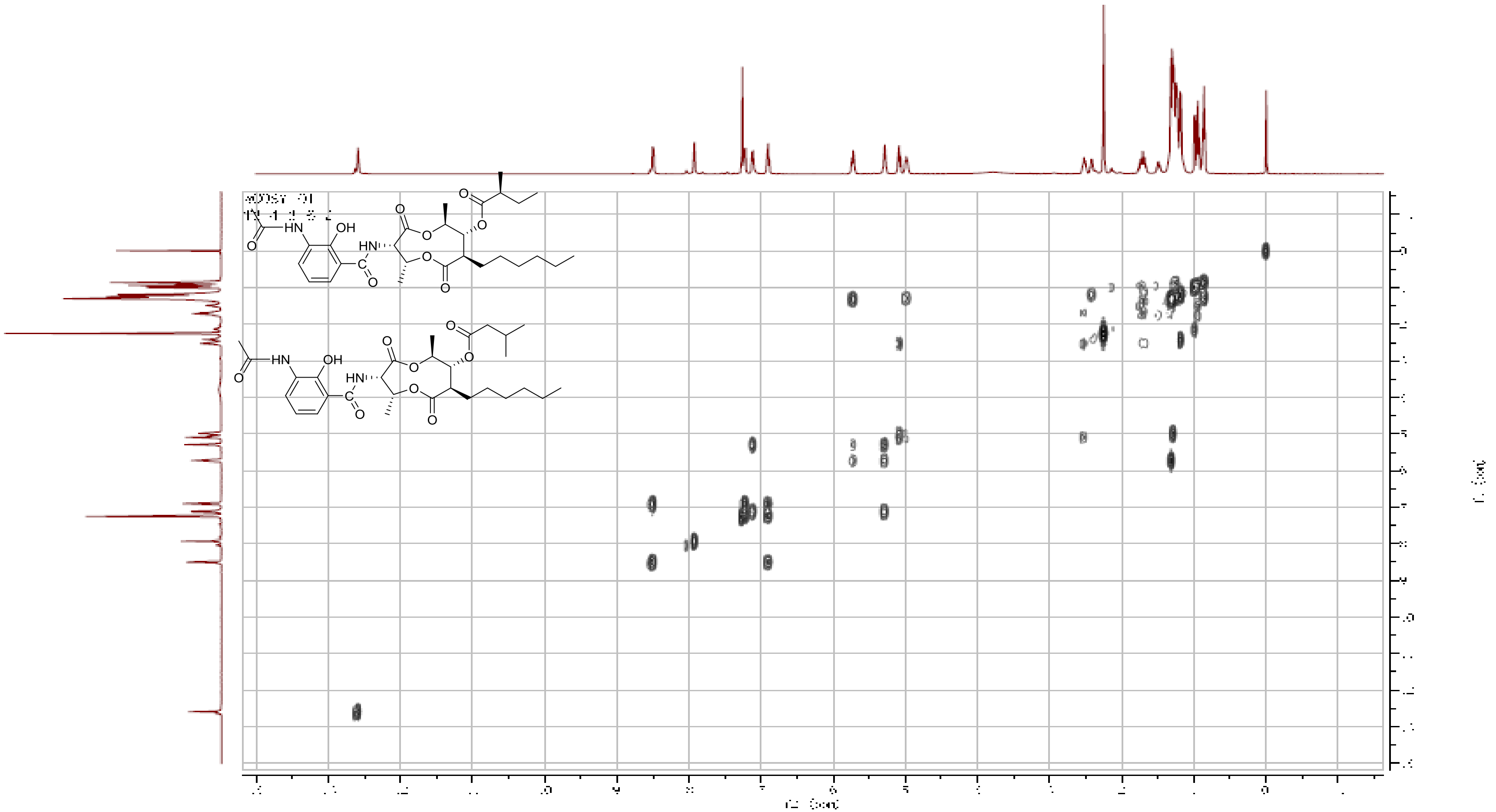

Fig. S34. ^{1}H-^{1}H COSY spectrum (500 MHz) of antimycin H (**4**) and NADA (**5**) in $CDCl_3$.

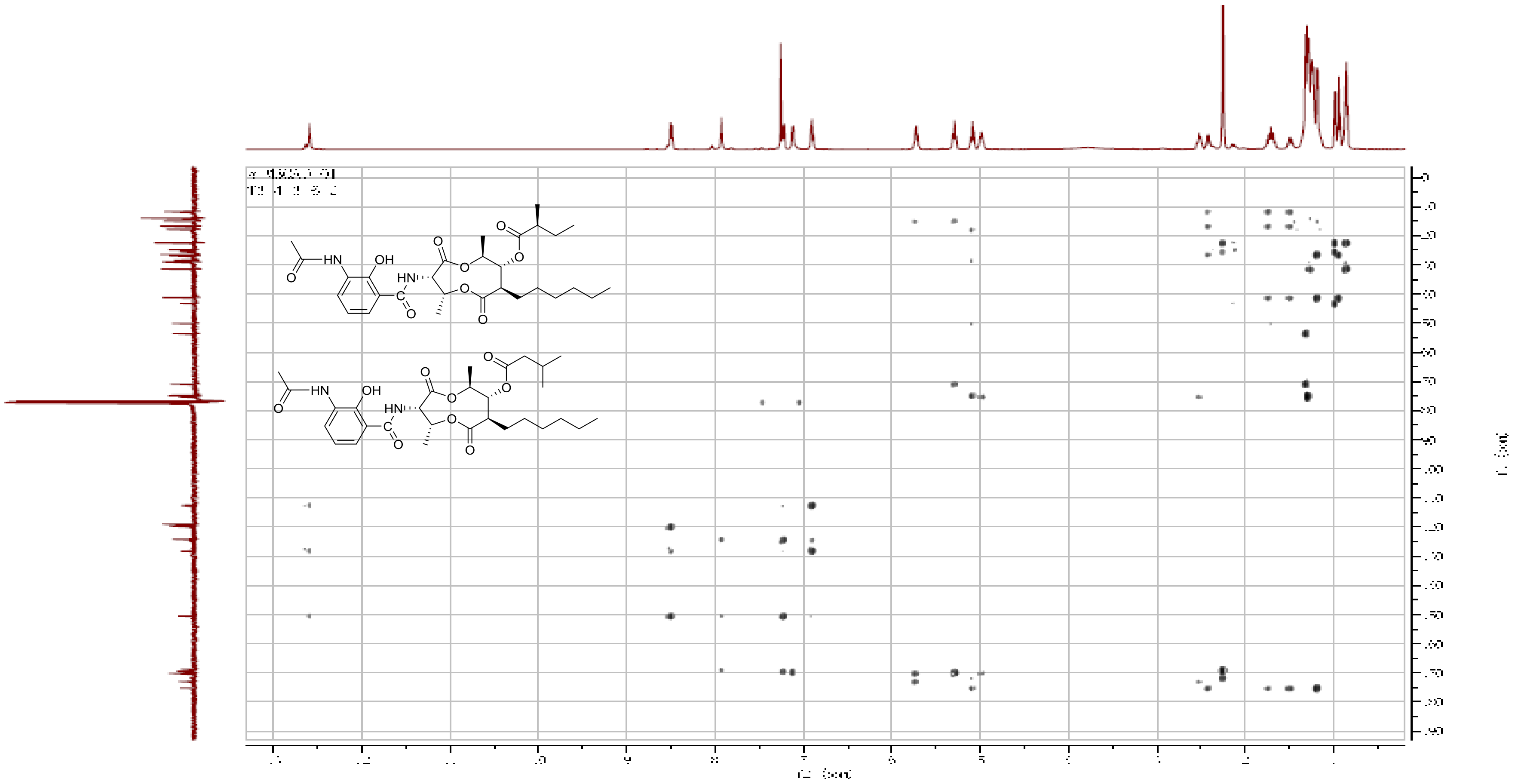

Fig. S35. HMBC spectrum (500 MHz) of antimycin H (**4**) and NADA (**5**) in $CDCl_3$

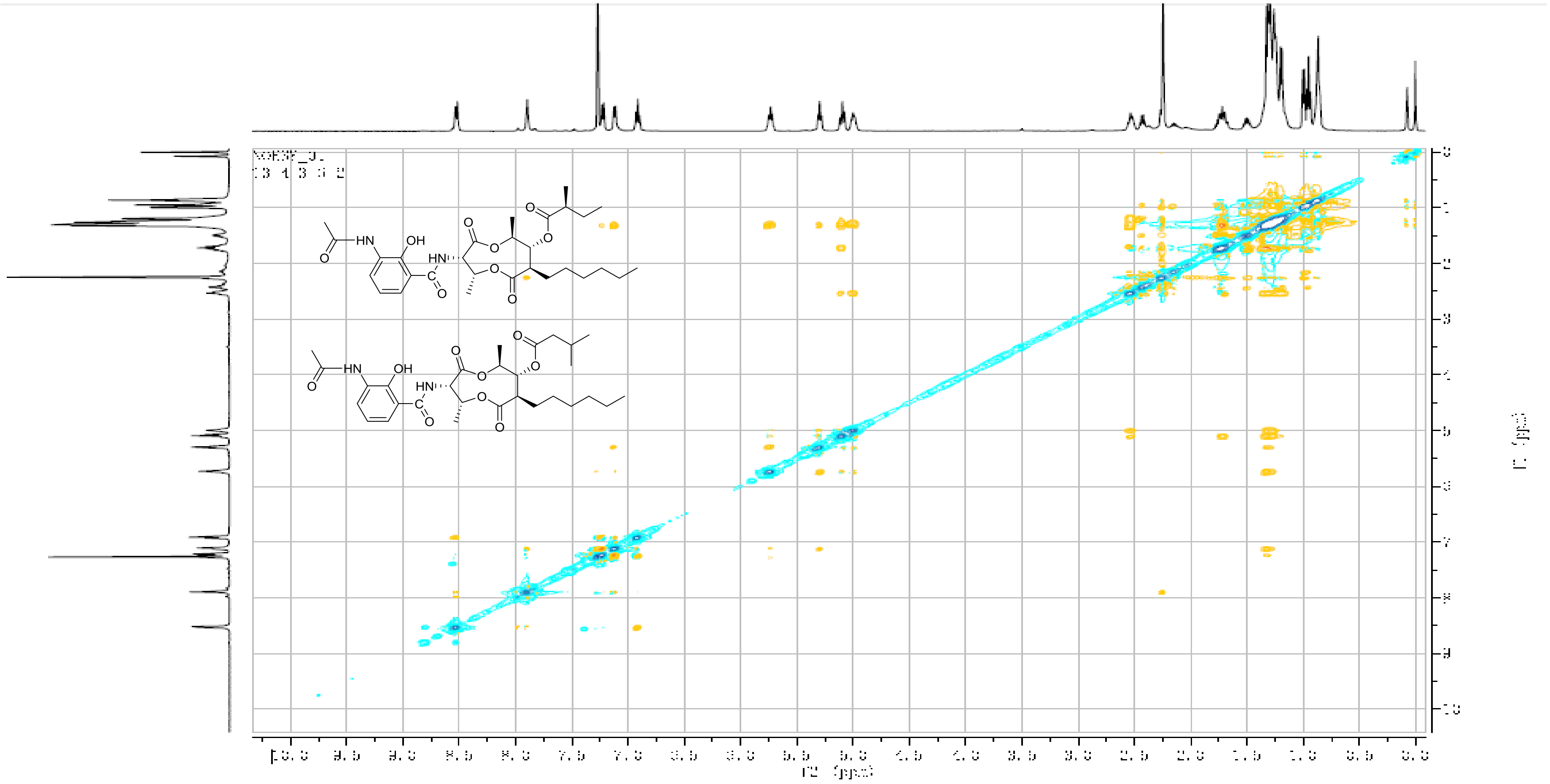

Fig. S36. NOESY spectrum (500 MHz) of antimycin H (**4**) and NADA (**5**) in $CDCl_3$
